# Supplementary material for: rec-YnH enables simultaneous many-by-many detection of direct protein–protein and protein–RNA interactions
Source: Nat Commun. 2018 Sep 14;9:3747. doi: 10.1038/s41467-018-06128-x (PMC6138660; doi:10.1038/s41467-018-06128-x)
Supplement: Supplementary file 1 — Supplementary Information [file 41467_2018_6128_MOESM1_ESM.pdf]

rec-YnH enables simultaneous many-by-many detection of direct protein-protein and protein-RNA interactions

Yang et al.

Supplementary Figures

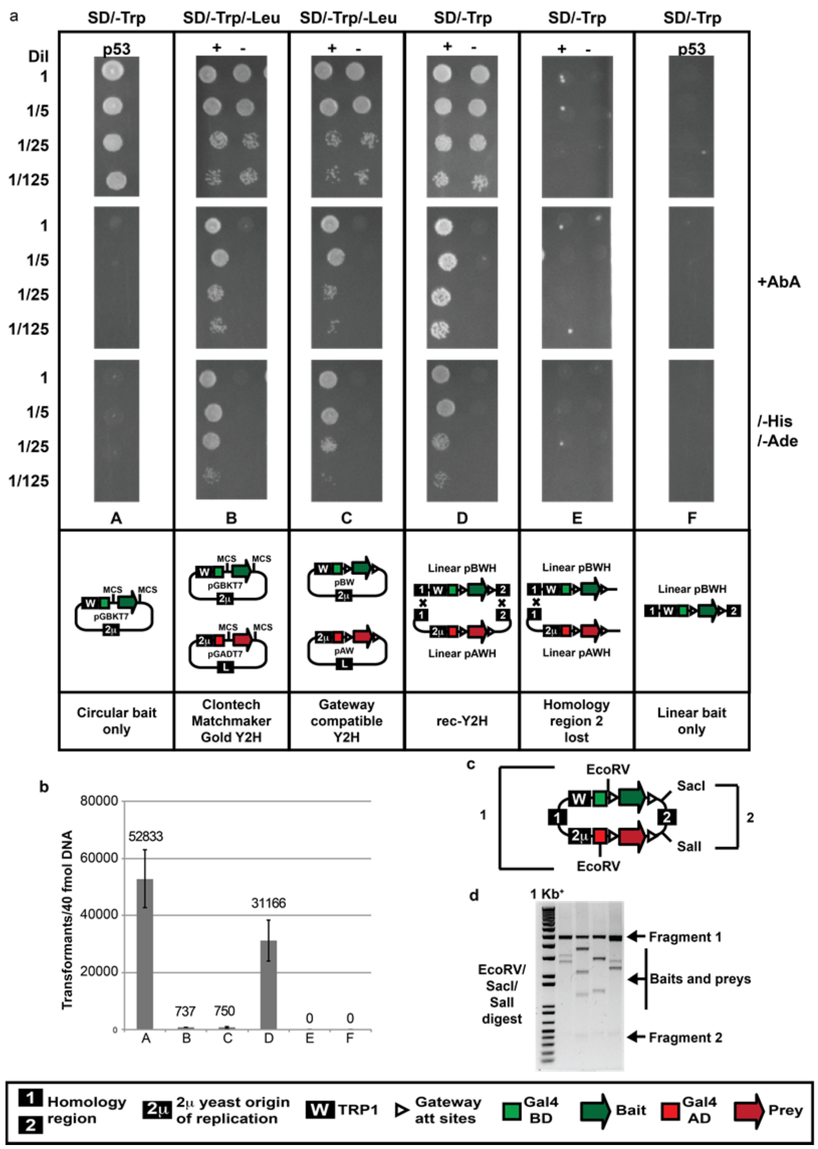

Supplementary Fig. 1. rec-Y2H versus traditional Y2H. (a) Forty fmol of the indicated (bottom) single plasmid (A and F), or 20 fmol of each plasmid (B-E) with a positive control pair (plus symbol: p53/largeT) or a negative control pair (minus symbol: p53/Lam) were transformed into competent Y2HGold cells. A. Clontech Matchmaker Gold Y2H bait vector. B. Clontech Matchmaker Gold Y2H vectors. C. Y2H vectors with Gateway cassette. D. rec-Y2H vectors with Gateway cassette, digested with I-SceI and I-CeuI to expose Homology regions 1 and 2. E. rec-Y2H vectors with Gateway cassette, digested with I-SceI and PI-PspI, leaving homology region 1 exposed, but eliminating homology region 2. F. rec-Y2H bait vector, digested with I-SceI and I-CeuI. See **Table S1** for a full description of all vectors. Serial dilutions (left) of transformed yeast cells were spotted on the indicated dropout selection agar plates (top and right) and grown for 3 days at 30°C. (**b**) Serial dilutions of transformed yeast cells (as in a) were spread on 10-cm SD/-Trp plates (A, D, E and F) or SD/-Trp/-Leu plates (B and C), and the number of colonies from each transformation was counted. **(c)** Schematic representation of pFAB, the recombination product of pBWH and pAWH after vector linearization by homing digestion (as in D) and transformation in yeast. Restriction enzymes sites are shown. **(d)** Plasmid DNA from four yeast colonies of one rec-Y2H X163 screen was extracted, and transformed into competent bacteria cells. Then, plasmid DNA was prepared and digested with EcoRV, SalI and SacI. For any bait-prey pair, fragment 1 and fragment 2 should have a constant size, showing proper recombination at Homology region 1 and 2. In addition, a different set of bands, corresponding to different inserts, appears for each bait-prey pair.

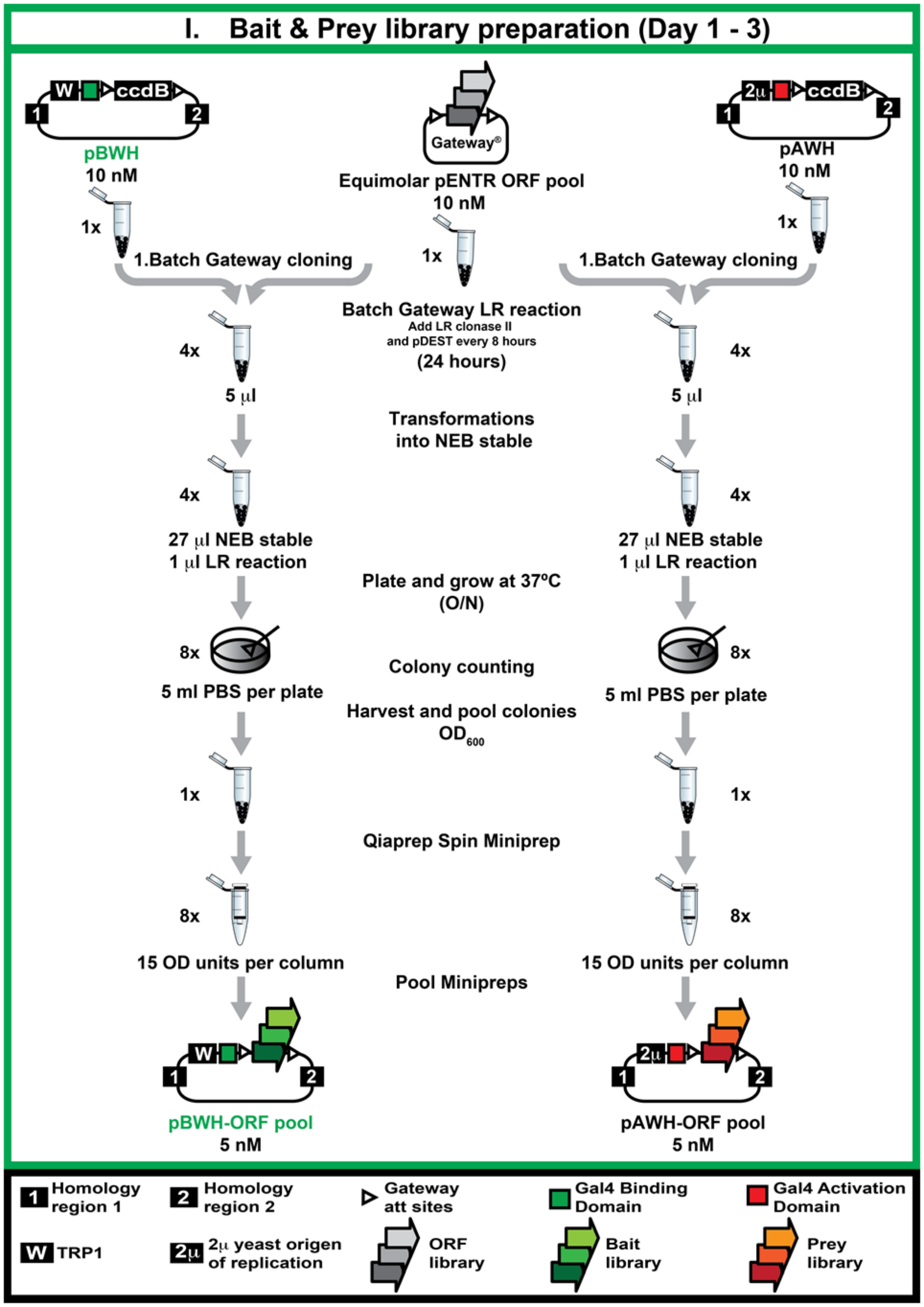

Supplementary Fig. 2. I. Bait & prey library preparation (Day 1 – 3) overview. A library of ORFs cloned into pENTR (entry vector for Gateway cloning) is transferred to pBWH and pAWH (pDEST bait and prey rec-Y2H vectors, respectively) by Gateway cloning. See **Table S1** for a full description of all vectors. Briefly, for each vector, four LR reactions are set up by mixing pAWH/pBWH, pENTR ORF pool and LR clonase II. Every 8 hours, pAWH/pBWH and LR clonase II are added again, for a total of three rounds (24 h). Reactions are stopped by incubating with Proteinase K. Each LR reaction is transformed into NEB stable cells, and each transformation is spread onto two LB-agar plates with the corresponding antibiotic. Colonies are harvested by adding 5 ml of PBS to each plate, and scraping off the colony-PBS emulsion. For each vector, the OD_{600} of the pool of colonies is measured, and DNA is prepared using eight Qiaprep Spin Miniprep columns. DNA from the eight columns is mixed together and diluted to 5 nM to obtain a pBWH-ORF pool and a pAWH-ORF pool. pAWH-ORF pools can also be used in rec-Y3H screening.

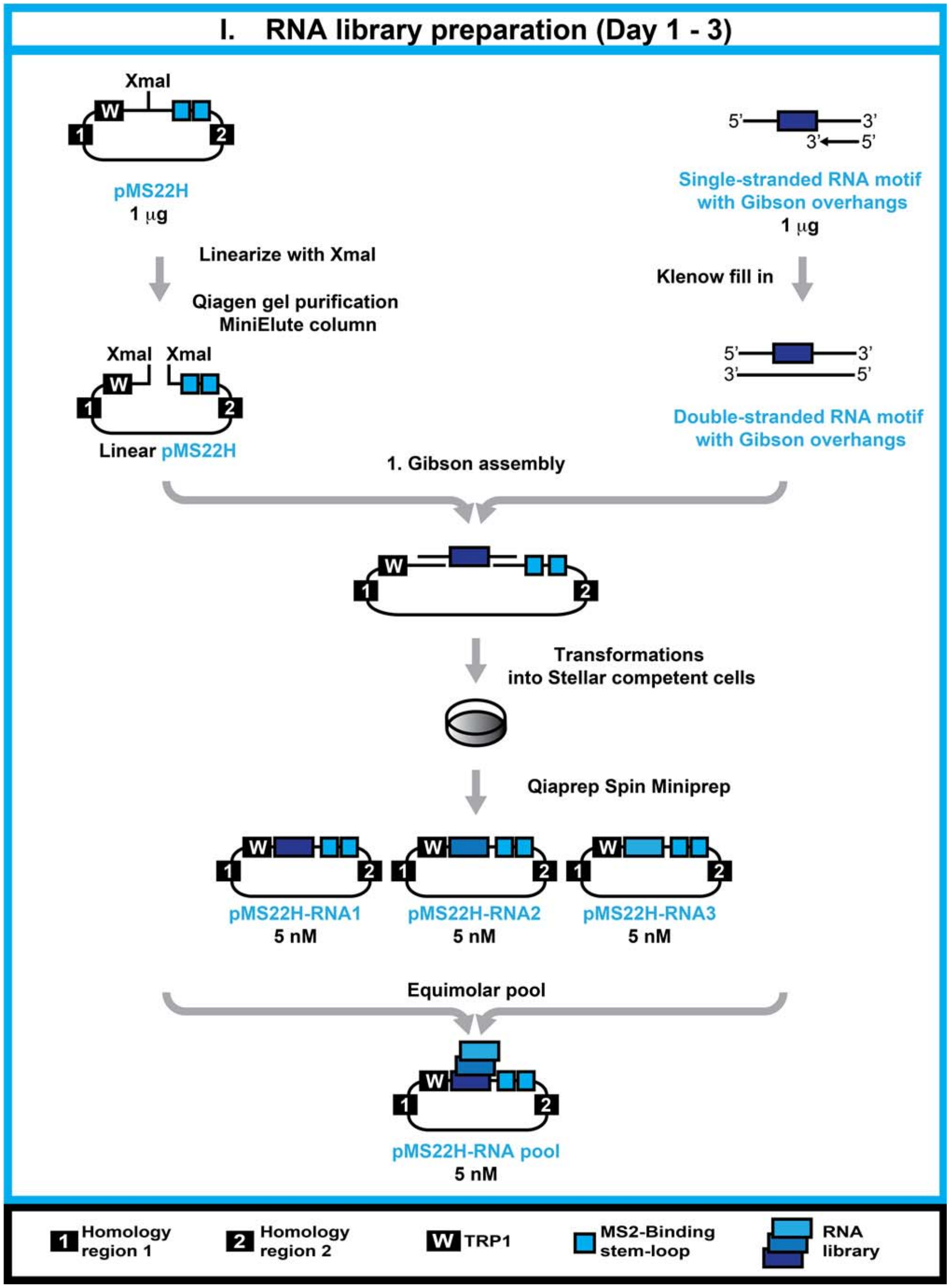

Supplementary Fig. 3. I. RNA library preparation (Day 1 – 3) overview. pMS22H (see **Table S1** for full description) is linearized at the XmaI site and gel purified. RNA motifs are ordered as single-stranded DNA oligonucleotides with Gibson overhangs. For each RNA motif, double-stranded DNA is generated through a Klenow fill-in reaction with the Oligo_gib_rev primer. Double-stranded RNA motifs and linear pMS22H are combined in a Gibson reaction mix for 1 hour at 50°C. Then, the mix is transformed into Stellar competent cells. For each RNA motif, one colony is picked and grown overnight in LB-Antibiotic (see **Table S2**), and DNA prepared with a Qiaprep Spin Miniprep column. Each pMS22H-RNA clone is diluted to 5 nM, and equal amounts of each are mixed to obtain a pMS22H-RNA pool. The pMS22H-RNA pool is used in combination with the pAWH-ORF pool (see **Supplementary Fig. 2**) for rec-Y3H screening.

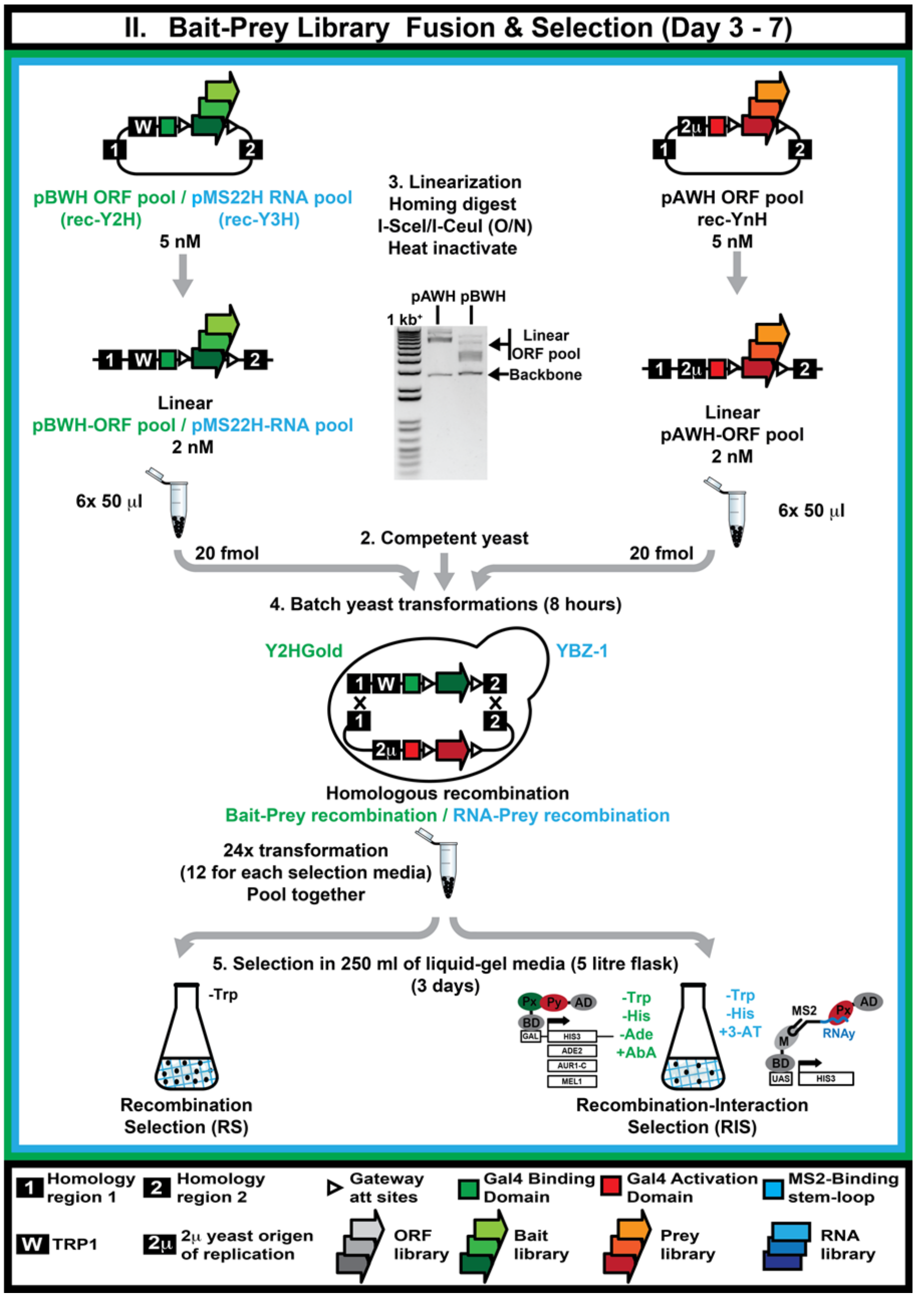

Supplementary Fig. 4. II. Bait-prey library fusion & selection for rec-YnH screening (Day 3 – 7) overview. Although the protocol for rec-Y2H is depicted in this figure, rec-Y3H follows the same steps with only minor differences (see **Table S5**). Reagents exclusively used in rec-Y2H and rec-Y3H are shown in green and blue, respectively. Common reagents are shown in black. Briefly, the pBWH-ORF pool (pMS22H-RNA pool for rec-Y3H) and pAWH-ORF pool are linearized by digestion with two homing enzymes (I-CeuI and I-SceI). A typical agarose gel of the resulting product is shown. Linear bait and prey pools are then co-transformed into competent Y2HGold cells (YBZ-1 for rec-Y3H). For simplicity, only the symbolic green protein-bait library is shown here. A pool of 24 small-scale transformations is split in half and added to 250 ml of each liquid-gel media (RS or RIS, see **Table S2** for media composition). Growth and selection of Y2H/Y3H interactions is done at 30°C for 60 hours. Only cells undergoing homologous recombination between a linear bait and a linear prey fragment at homology regions 1 and 2 give rise to a vector containing both the TRP1 marker and a yeast origin of replication. As such, only these cells can propagate in -Trp medium.

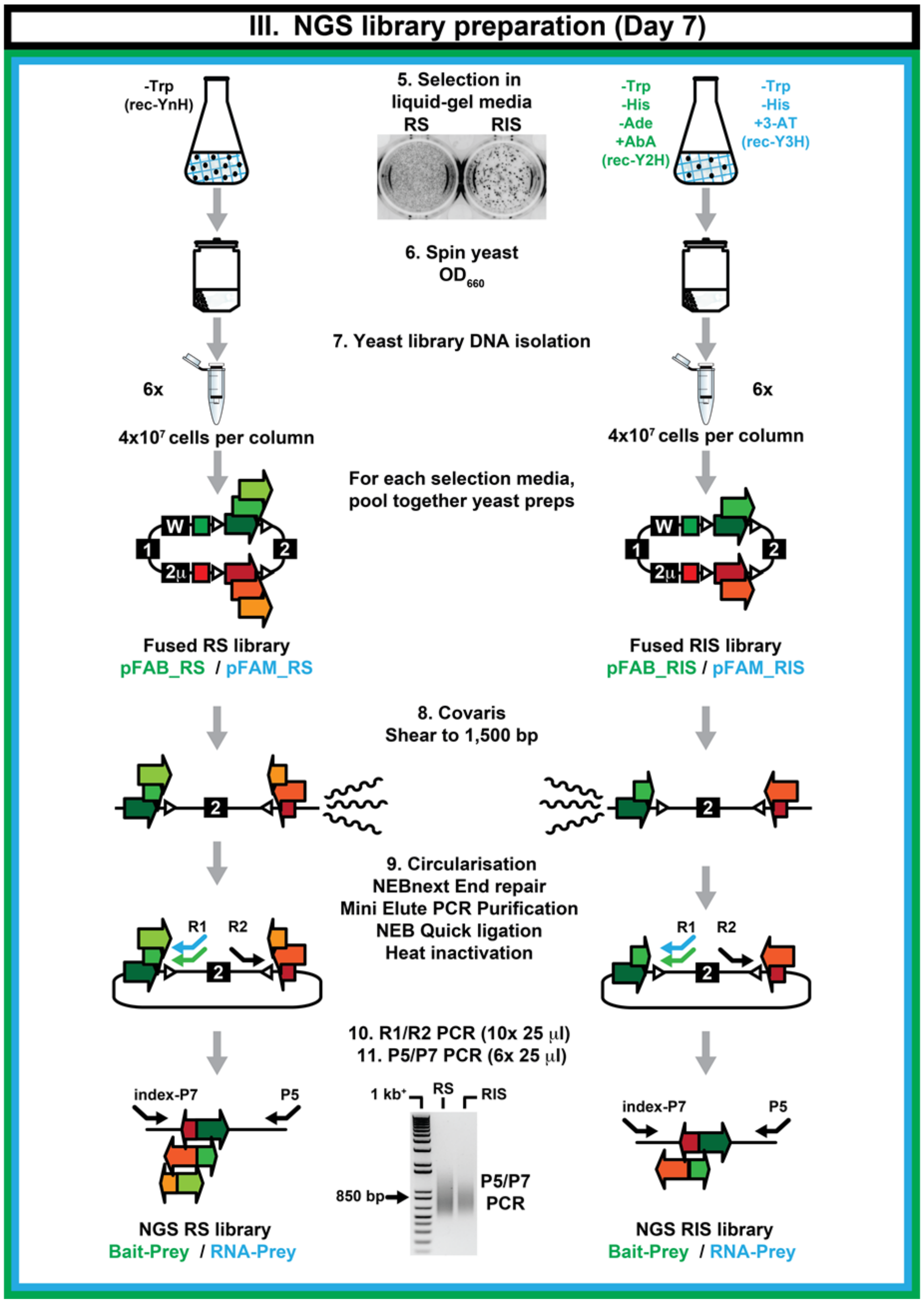

Supplementary Fig. 5. III: NGS library preparation for rec-YnH screening (Day 7) overview. Although the rec-Y2H protocol is depicted in this figure, rec-Y3H follows the same steps with only minor differences (green: rec-Y2H, blue: rec-Y3H, see **Table S5** for details). The top image shows a typical example of colonies suspended in recombination-selection (RS) and recombination-interaction-selection (RIS) liquid-gel media (top view). For each selection media, colonies are harvested from the liquid-gel cultures by centrifugation, and library DNA is extracted (pFAB for rec-Y2H and pFAM for rec-Y3H). DNA is sheared to 1,500 bp by Covaris, and circularised by intramolecular ligation. Fragmentation of DNA happens randomly. Only fragments containing both bait and prey are shown, however, other DNA fragments from the plasmid are also present in this mix. In a first PCR step, circular DNAs containing both a 3' end of a bait and a prey are selectively amplified, and R1 and R2 Illumina adaptors are added. A second PCR reaction introduces an index sequence for multiplexing and P5/P7 Illumina attachment sequences. The bottom picture shows a typical gel of the NGS libraries obtained from both RS and RIS media.

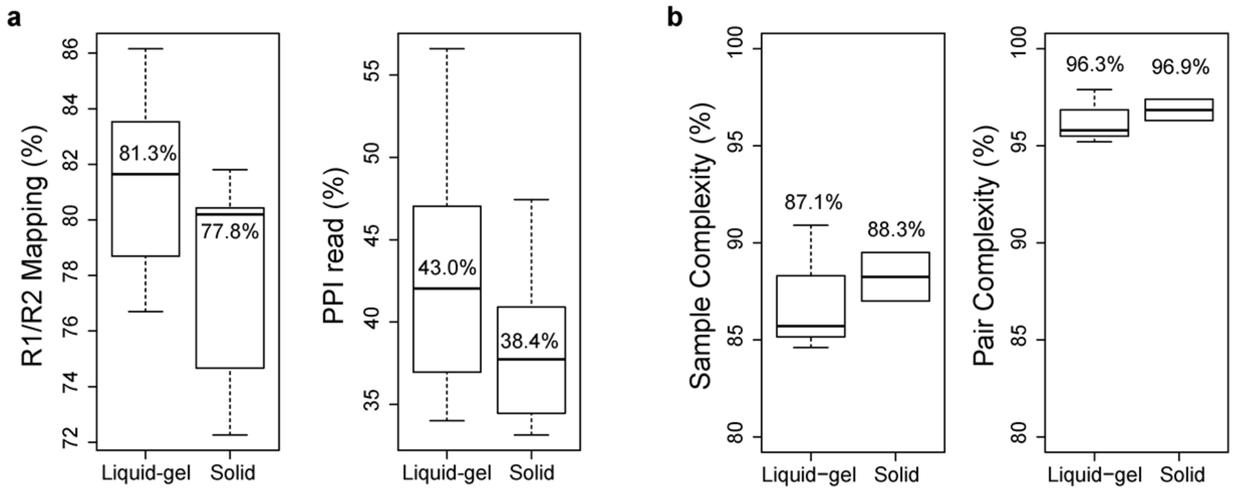

Supplementary Fig. 6: Comparison of liquid-gel and agar plate culture (a) Estimation of rec-Y2H read mapping efficiency according to culture conditions. On average, 81.3% and 77.8% of total reads are mapped with R1/R2 PCR primers, and 43.0% and 38.4% of reads are usable to map interaction pairs for liquid-gel and plate culture, respectively. The liquid-gel culture gives better mapping efficiency but it was not statistically significant considered the small number of experiments (see **Supplementary Data 2**). (**b**) Estimation of rec-Y2H sample and pair complexity according to culture conditions. On average, 87.1% and 88.3% of sample complexity, and 96.3% and 96.9% of pair complexity were estimated for liquid-gel and plate culture, respectively. There were no statistical differences. The box of the box-plot indicates the median, upper and lower quartiles for each value distribution.

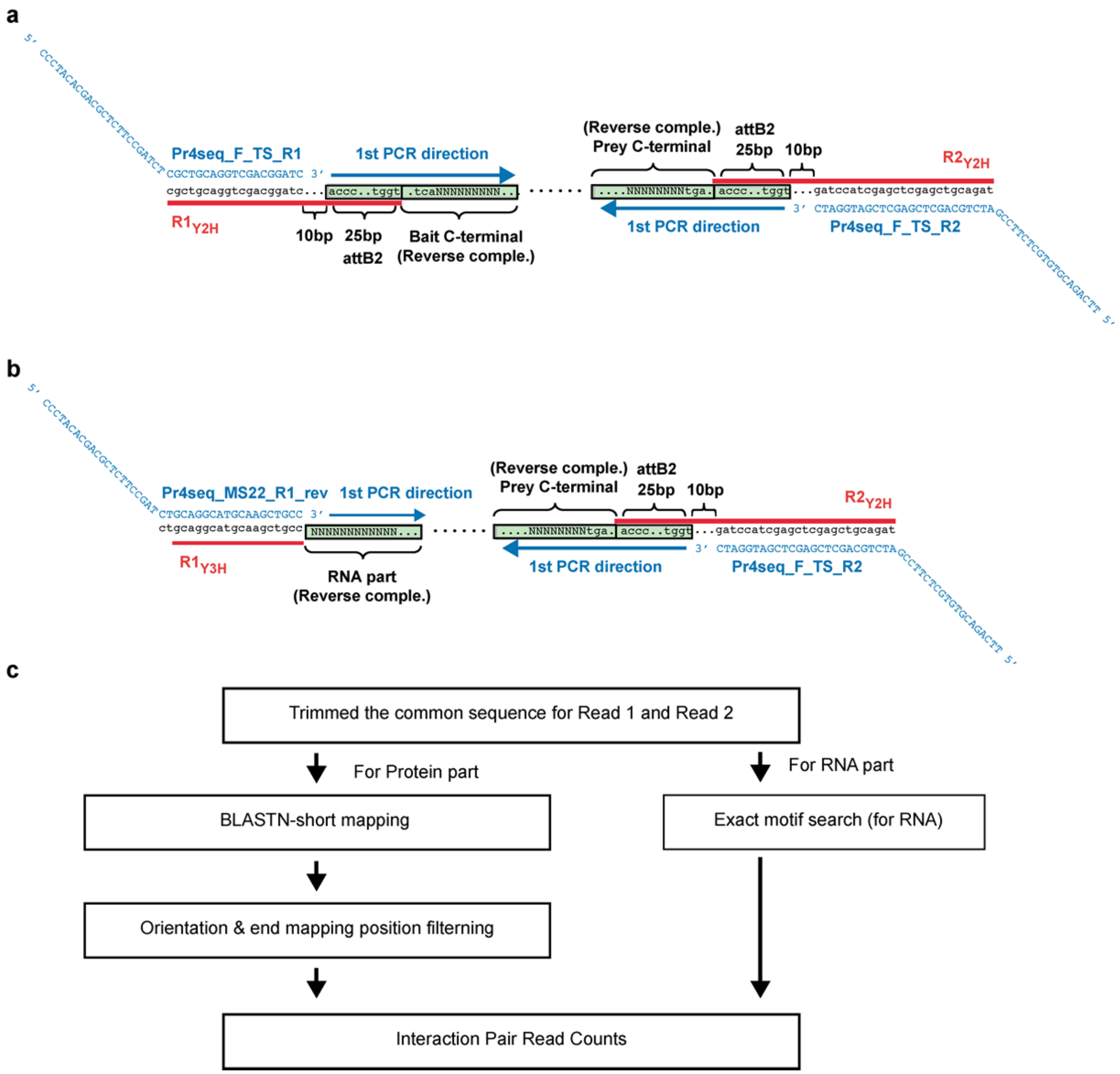

Supplementary Fig. 7: Detailed view of library sequencing scheme and mapping pipeline. (a) For the rec-Y2H library, circular DNA containing both the 3’ end of a bait and a prey are specifically amplified among the pool of fragments by a first PCR step, and R1 and R2 Illumina adaptors are simultaneously added. A second PCR reaction introduces an index sequence for multiplexing and P5/P7 Illumina attachment sequences. (**b**) The rec-Y3H library is amplified the same way except by using Pr4seq_MS22_R1_rev primer instead of Pr4Seq_F_TS_R1. (**a,b**) The common adaptor sequences ($R1_{Y2H}$, $R2_{Y2H}$, and $R1_{Y3H}$) are depicted as red lines. NGS gives reverse complementary sequences of proteins and RNAs. (**c**) The common adaptor sequences are trimmed by the cutadapt software. Then the remaining sequences are mapped to an input library sequence file with blastn-short for rec-Y2H (see Methods). In the case of rec-Y3H, the RNA input sequences are directly mapped with exact sequence match.

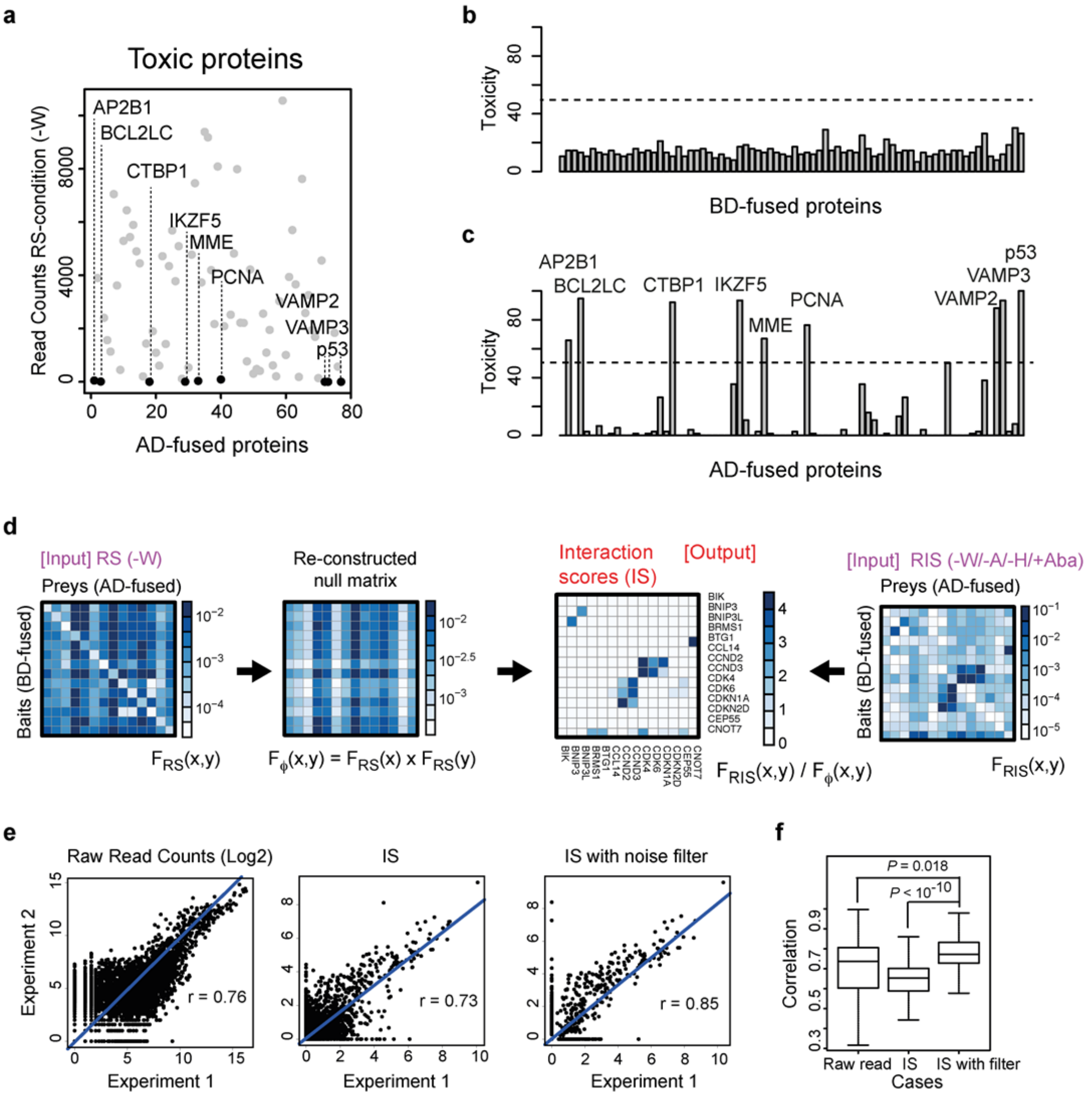

Supplementary Fig. 8. Tests for toxicity proteins, and comparison between IS and noise-filtered IS. BD-empty (pAWH X76/pBWH empty) or full (pAWH X76/pBWH X76) set was transformed into yeast and spread on RS and RIS plates. DNA was prepared for NGS sequencing as described in **Supplementary Fig. 5**. **(a)** Detection of toxic prey proteins by analysing the mapped read counts from the BD-empty screen on RS media. Dark circles indicate toxic proteins (very low read counts indicates toxicity)**. (b, c)** Toxicity was measured by the percentage of zero reads partners according to AD-fused or BD-fused proteins. Dashed line indicates 50%. **(b)** Toxicity of baits. Most of them have similar level of toxicity. On average 14.4% of the partners have zero reads count. **(c)** Toxicity of preys. The level of toxicity changes a lot depending on the preys. 9 of preys have over 50% of partners with zero reads counts. These 9 prey proteins are corresponding with toxic proteins in BD-empty screen in (**a**). **(d)** Computational analysis pipeline of rec-Y2H X71 used to produce the interaction score (IS) with a published method[1]. As a joint probability, the null matrix ($F\phi(x,y)$) was generated by multiplying the marginal probabilities of baits and preys in the RS media ($F_{RS}(x,y)$). The marginal probability of $F_{RS}(x)$ and $F_{RS}(y)$ indicates the row- and column-wise sum of the detection frequency $F_W(x,y)$, respectively. The signal from RIS media ($F_{RIS}(x,y)$) is normalized by the null matrix ($F\phi(x,y)$) to generate interaction scores (IS). (**e**) Correlation of signals between two full-screen replicates (starting at library transformation, see **Fig. 1**) with different methods. The noise-filtered IS (r = 0.85) shows a better correlation coefficient than the unfiltered IS (r = 0.73). (**f**). In all experiments, the noise-filtered IS gives the best correlation between experiments ($P < 10^{-10}$; two-tailed unpaired t-test). The box of the box-plot indicates the median, upper and lower quartiles for each population of correlation.

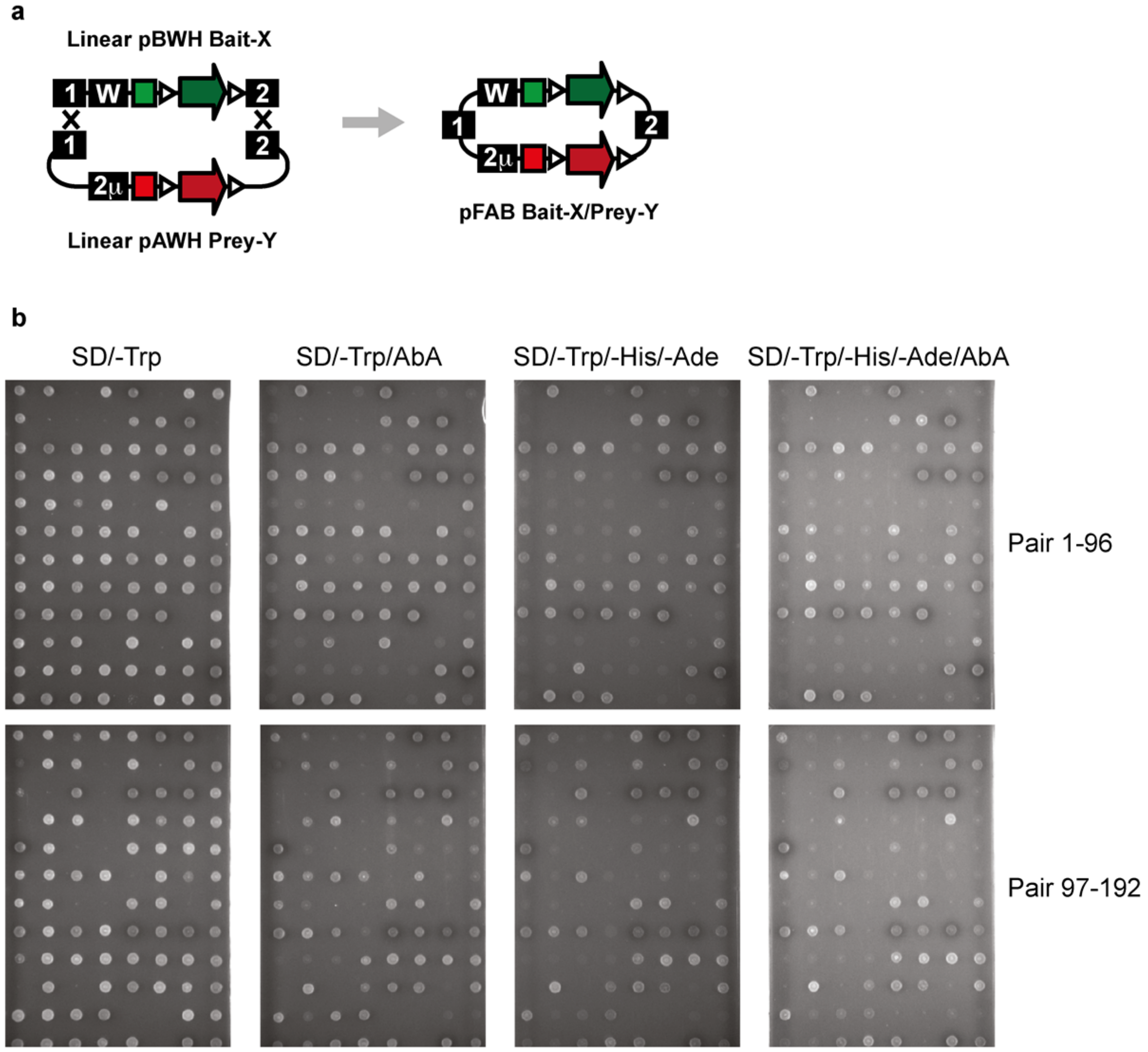

Supplementary Fig. 9. Representative spot test for selected X71 screening pairs. (a) General graphical representation of recombination between the linearized pBWH bait-X and pAWH prey-Y to produce a pFAB bait-prey fusion vector. **(b)** Four fmol of linear pBWH Bait-X and 4 fmol of linear pAWH Prey-Y were co-transformed into competent Y2HGold cells and spotted on SD/-Trp, SD/-Trp/AbA (low stringency), SD/-Trp/-His/-Ade (medium stringency) and SD/-Trp/-His/-Ade/AbA (high stringency) agar plates. Pairs that did not grow on SD/-Trp and false positives were retested. Pairs with ORFs with an incorrect identity (identified by restriction enzyme digest and PCR) were excluded from the performance analysis. See **Supplementary Data 4a** for a list of pairs and results.

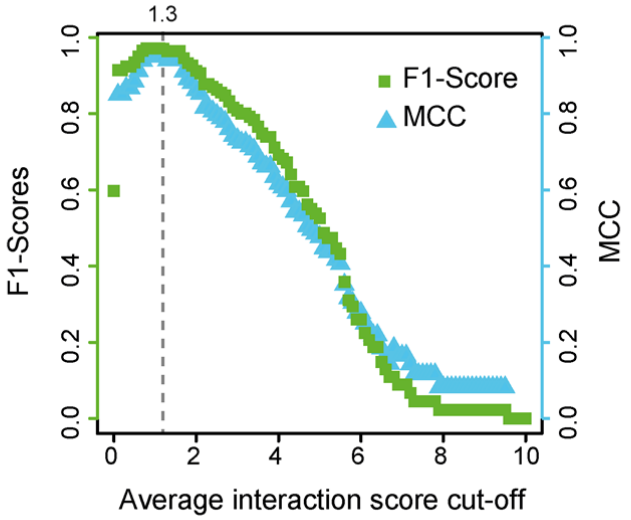

Supplementary Fig. 10. F1-Score and MCC distribution based on spot test results. Determination of the optimal interaction score cut-off based on the harmonic average of precision and sensitivity (F1-score, green) and Matthews's correlation coefficient (MCC, cyan). The performances were evaluated based on 207 individual spot tests for hetero-interactions.

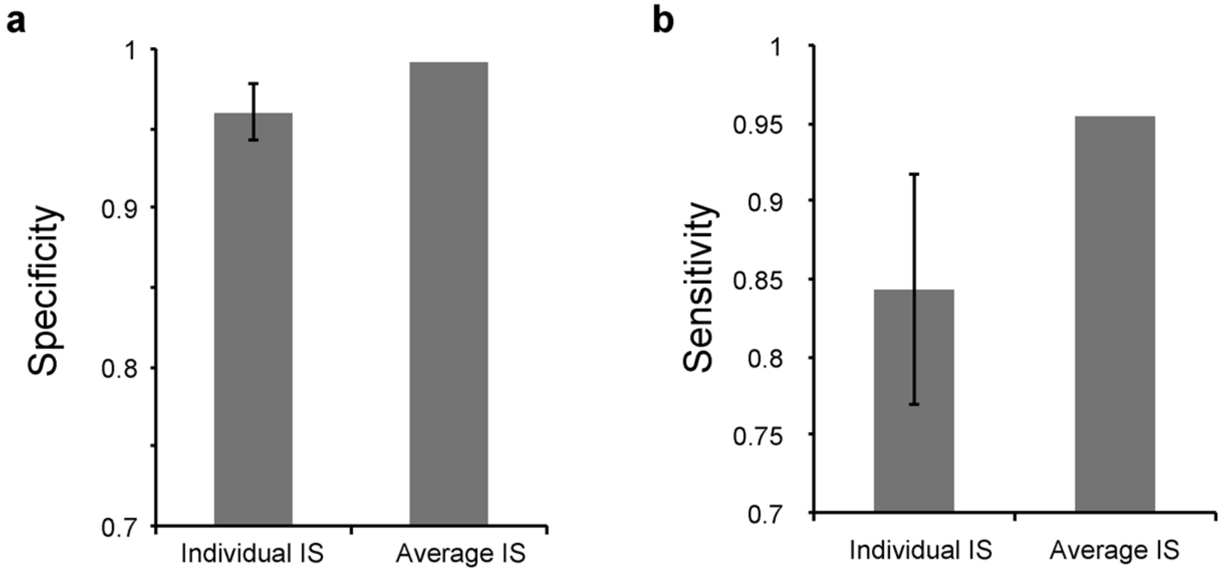

Supplementary Fig. 11. Sensitivity and specificity in individual and average experiments. This sensitivity and specificity plot of rec-Y2H is based on individual spot-test results in **Supplementary Fig. 9 (Supplementary Data 4a)**. We used the optimal IS cut-off based on the F1-Score (=1.2). (**a**) Specificity of individual experiments and average experiments. (**b**) Sensitivity of individual experiments and average experiments. (**a, b**) Error bars represent standard deviations.

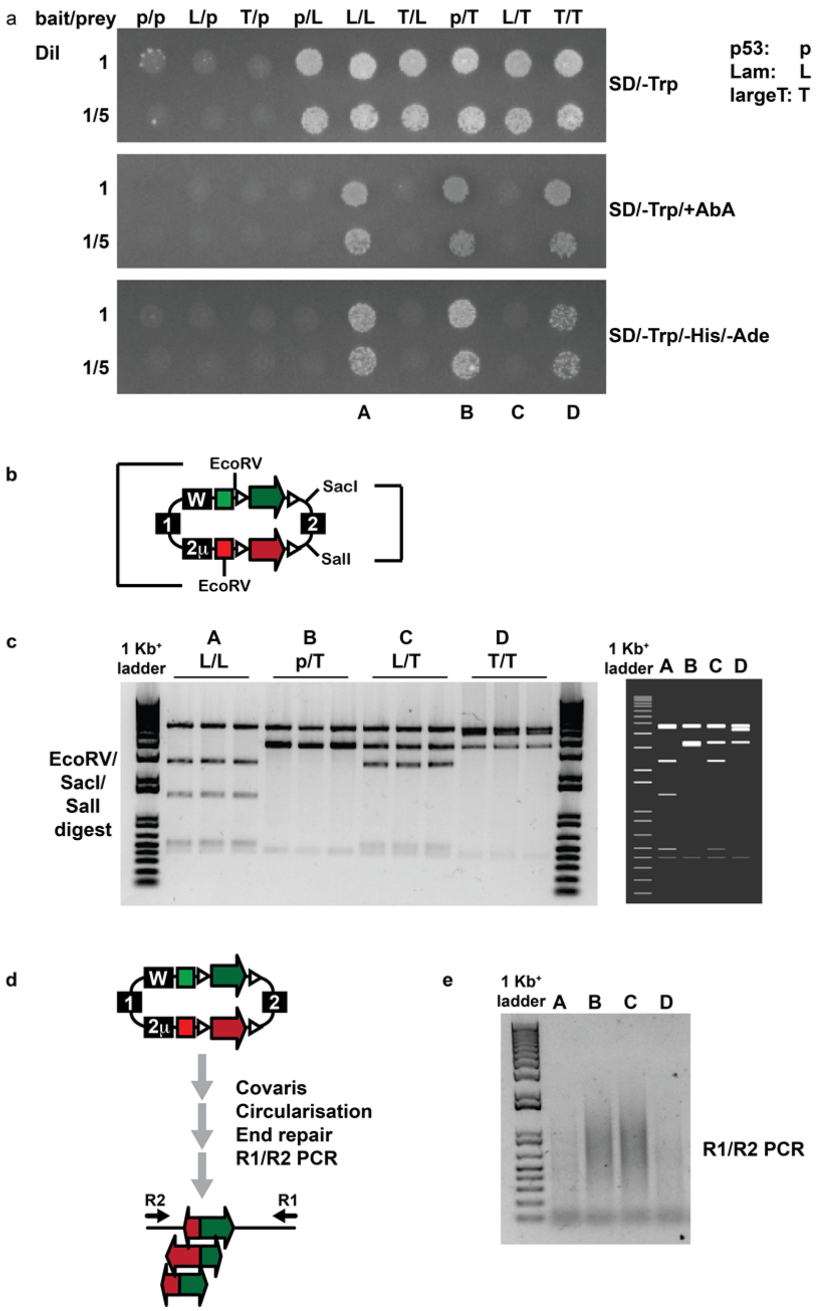

Supplementary Fig. 12. Homodimers versus heterodimers in rec-Y2H. (a) Twenty fmol of the indicated (top) linear bait and linear prey plasmids (p: p53, L: Lam, T: largeT) were co-transformed into competent Y2HGold cells, spotted on the indicated dropout selection agar plates (right) and grown for 3 days at 30°C. Transformed yeast cells from SD/-Trp plate from pairs A, B, C, D (bottom) were picked and used in subsequent experiments. (**b)** Schematic representation of pFAB, the recombination product of pBWH (bait vector) and pAWH (prey vector) after vector linearization by homing digestion and transformation in yeast. Restriction enzymes sites are shown. **(c)** Plasmid DNA from pairs A, B, C and D was extracted from yeast, and transformed into competent bacteria cells. Then, plasmid DNA was prepared from three different colonies and digested with EcoRV, SalI and SacI. For each pair, expected sizes are shown in the right panel, showing proper recombination at Homology region 1 and 2 in all cases. **(d)** Schematic representation of the preparation of R1/R2 PCR product. For each pair, pFAB was extracted from yeast, sheared by covaris, end repaired, circularized and amplified by 25 cycles of PCR with R1/R2 primers. **(e)** R1/R2 PCR products for pairs A, B, C and D. A and D are homodimer pairs. B and C are heterodimer pairs.

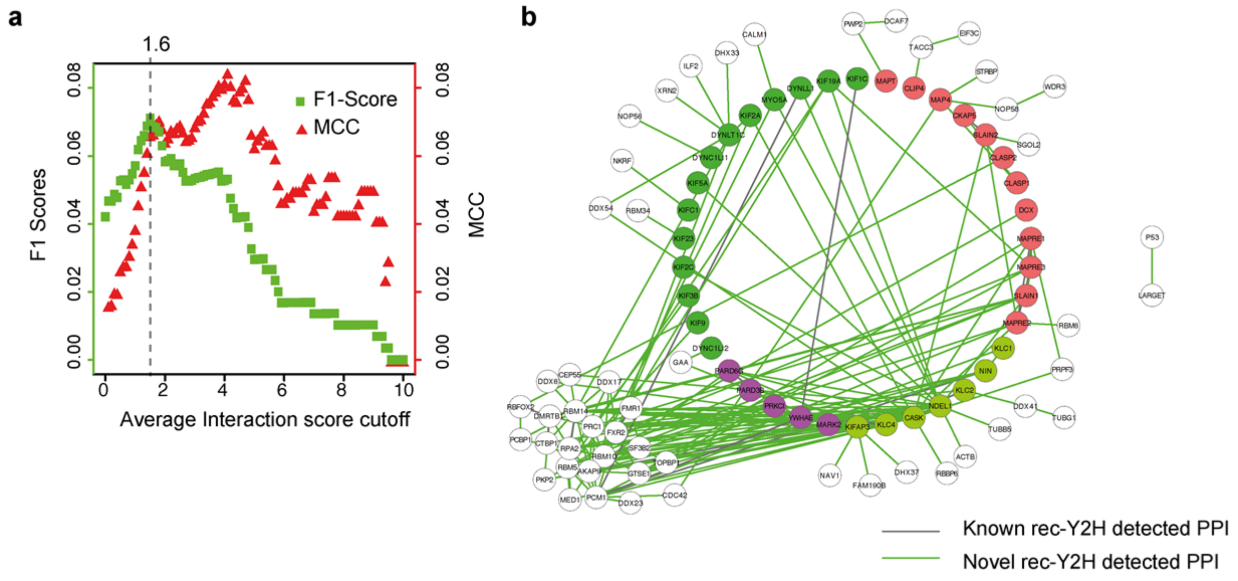

Supplementary Fig. 13. Optimal interaction score cut-off for the X163 set and interactions detected above cut-off. (**a**) The harmonic average of precision and sensitivity (F1-score) and Matthew´s correlation coefficient (MCC) plotted as a function of average IS cut-off. The performances were evaluated based on the BioGRID and HIPPIE PPI databases. We stringently defined all pairs not found in both databases as none-interacting pairs. (**b**) Connectivity plot showing all PPI-database-annotated and found interactions between PAR proteins, microtubule-associated proteins (MAPs), motor adaptors and motor proteins, and other proteins present in the X163 library. Violet=Par-proteins, bright green=motor protein adaptors, dark green=motor proteins, red=microtubule associated proteins.

a

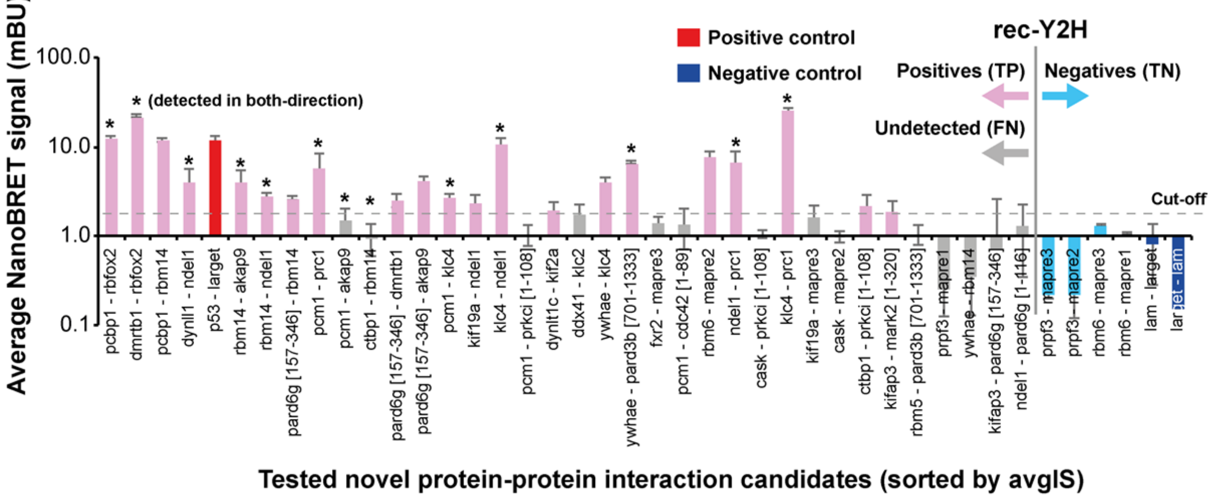

b

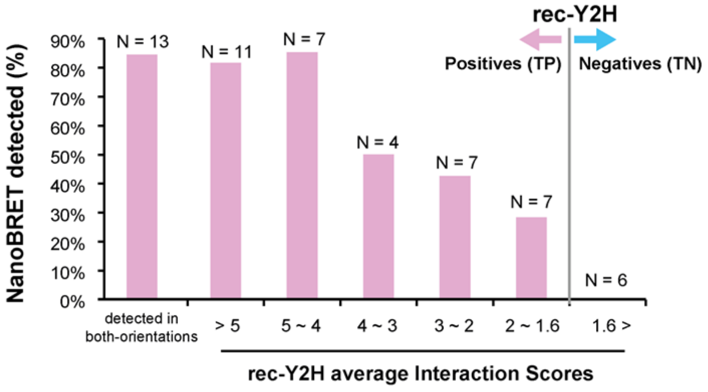

Supplementary Fig. 14. NanoBRET protein-protein interaction validation results. (**a**) NanoBRET validation experiment results. Tested protein-protein pairs were sorted by their average IS, decreasing from left to right. Red and blue bars represent positive and negative interaction controls, respectively. Pink and grey bars represent rec-Y2H positive interactions. Pink bars=above NanoBRET cut-off, grey bars = below NanoBRET cut-off. Light blue bars are rec-Y2H negative interactions. The cut-off was defined by mean plus 2.3 standard deviations of NanoBRET scores of all negative interactions. Asterisk (*) indicates interactions detected above the cut-off (average IS=1.6) in both orientations (Px-AD – Py-BD or vice versa). Average of background corrected NanoBRET signals are shown, error bars represent standard deviations. (**b**) The percentages of NanoBRET-tested interaction pairs sorted by categories. rec-Y2H scores were binned into 5 different groups. The number of cases in each group has shown in the top of the bar graph. The negative control and rec-Y2H negative interactions contained no NanoBRET positive pairs.

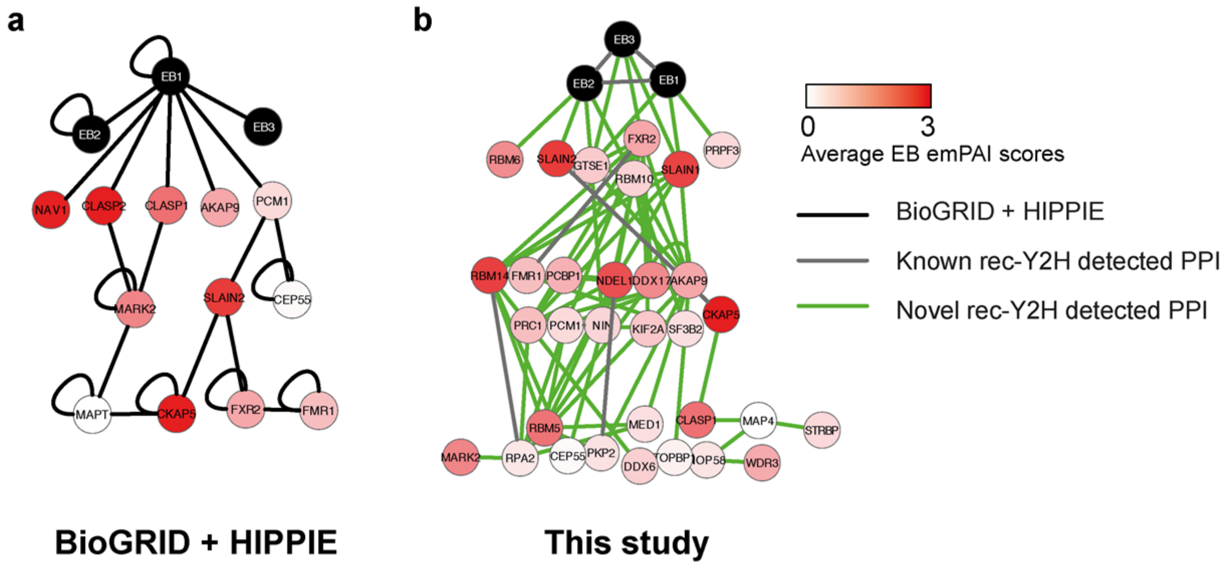

Supplementary Fig. 15. Dissecting the co-precipitated complexes of microtubule-cytoskeleton regulators with binary physical interactions. (**a**) BioGRID and HIPPIE database-annotated physical interactions. (**b**) Physical interactions found in this study. Among them BioGRID and HIPPIE annotated interactions are shown as grey line (Known rec-Y2H detected PPI). (**a, b**) The node colours correspond to the average pull-down emPAI scores for all EBs. Out of the 98 +TIP proteins tested, using interactions annotated in known PPI databases allows mapping of interactions of 12 EB-interacting proteins (a), while rec-Y2H enabled us to map the direct and indirect interactions of 32 proteins (b).

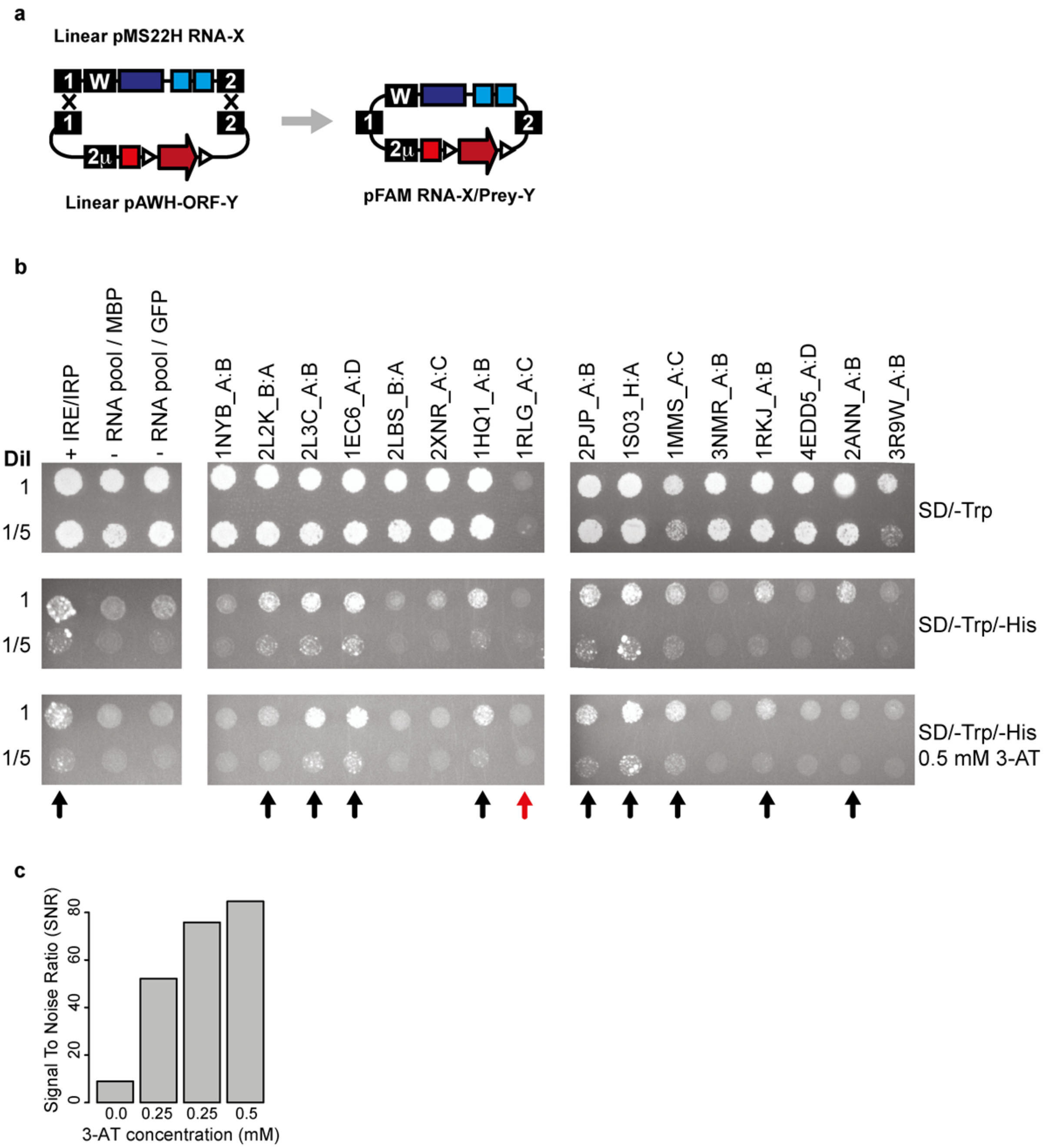

Supplementary Fig. 16. Spot test for published co-complex structures of protein domain-RNA pairs of the RNAlib library (a) Graphical representation of recombination between linearized pMS22H-RNA-X and pAWH-Prey-Y to produce a pFAM-RNA-prey fusion vector. **(b)** Ten fmol of linear pMS22H-RNA and 10 fmol of linear pAWH-Prey were co-transformed into competent YBZ-1 cells and spotted on SD/-Trp. After 3 days growth at 30°C, spots were replicated on SD/-Trp/-His and SD/-Trp/-His + 0.5 mM 3-AT. Plus symbol: IRP/IRE positive control. Minus symbol: pAWH with MBP or GFP proteins (considered non-RNA-binding proteins) co-transformed together with a pool of 16 RNAs, showing that none of the RNAs screened have auto-activating activity. Black arrows: positive interactions. Red arrow: no growth on SD/-Trp indicates toxicity of either the RNA or ORF in the 1RLG_A:C pair. See **Supplementary Data 7** for input sequences. (**c**) Signal to noise ratios according to 3-AT concentration on the medium. Signals were average IS scores from expected positive pairs (diagonal line in **Fig. 6a**) and noise were average IS scores of the all other pairs from off-diagonal lines.

a

```
>dsRBM2 (wt)
MPSGKNPVMILNELRPGLKYDFLSESGESHAKSFVMSVVVDGQFFEGSGRNKKLAKARAAQSALATVFNLHL

>dsRBM2 mut (S29A;H30A)
MPSGKNPVMILNELRPGLKYDFLSESGEAAAKSFVMSVVVDGQFFEGSGRNKKLAKARAAQSALATVFNLHL
```

```
>dsRBM1 (wt)
MPGPVLPKNALMQLNEIKPGLQYMLLSQTGPVHAPLFVMSVEVNGQVFEGSGPTKKKAKLHAAEKALRSFVQFPN

>dsRBM1 mut (E16A)
MPGPVLPKNALMQLNAIKPGLQYMLLSQTGPVHAPLFVMSVEVNGQVFEGSGPTKKKAKLHAAEKALRSFVQFPN
```

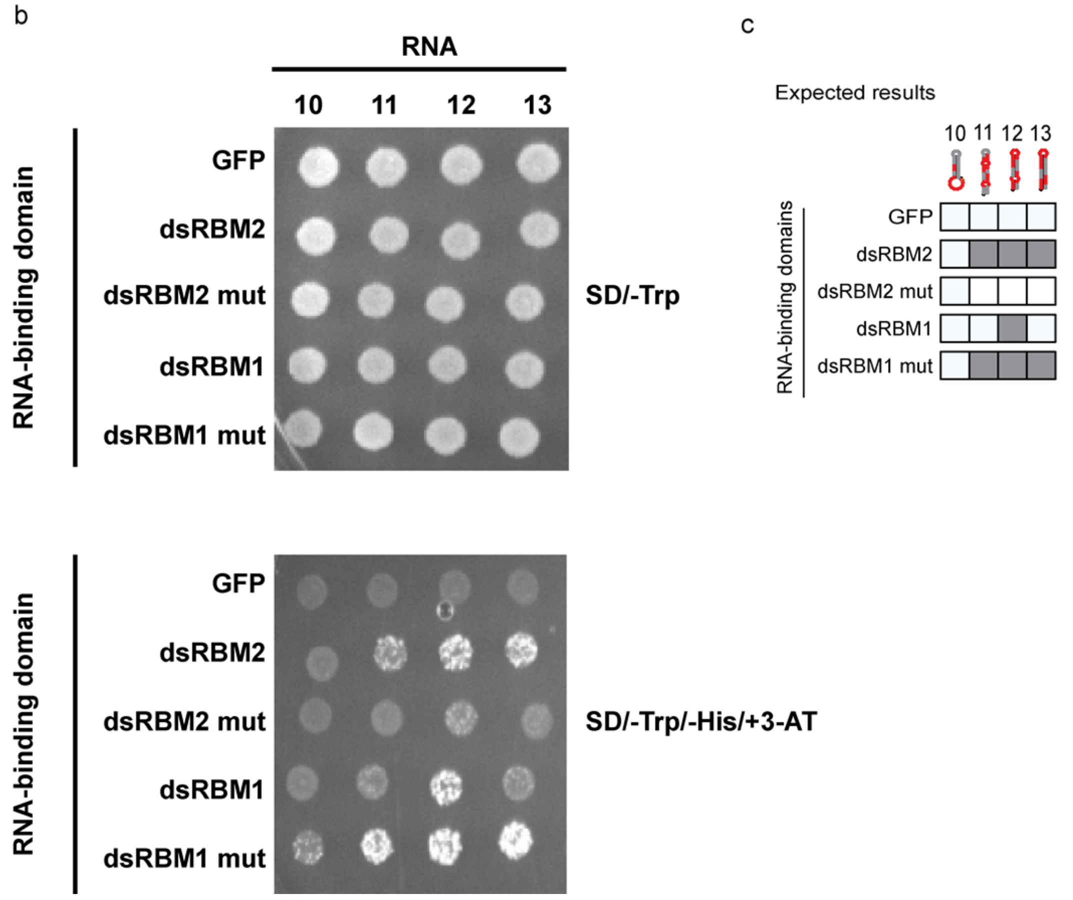

Supplementary Fig. 17. Mutations affecting dsRBM2 and dsRBM1 RNA binding specificity and overall activity (a) Mutated amino acids. **(b)** 10 fmol of linear pMS22H-RNA and 10 fmol of linear pAWH-Prey were co-transformed into competent YBZ-1 cells and spotted on SD/-Trp and SD/-Trp/-His +1 mM 3-AT and grown for 4 days. See **Supplementary Data 7** for input sequences. (**c**) Interaction matrix showing the obtained results with grey filled boxes representing interactions.

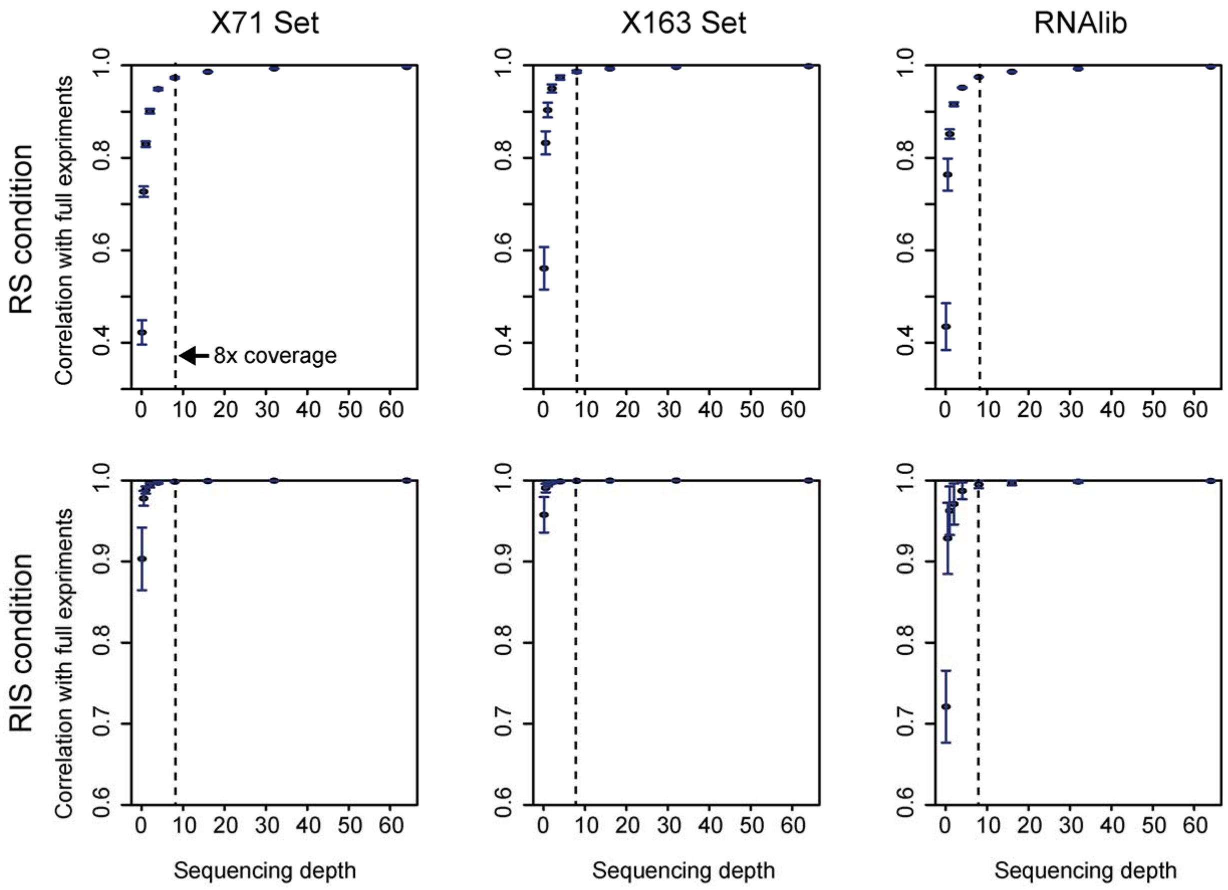

Supplementary Fig. 18. Computational saturation test by sub-sampling of sequencing reads. Correlation between frequency matrixes from sub-sets and full data sets. The Pearson correlation coefficients are shown on the Y-axis according to the sub-set size tested. The tested sub-set sizes (sequencing depth) were 0.1x, 0.5x, 1x, 2x, 4x, 8x, 16x, 32x, and 64x. 1x means that the number of read pairs used is identical to the number of theoretical combinations. For example, 323 pair reads would correspond to 1x (17x19) for an RNAlib screen. With 8x coverage, correlation coefficients of 0.97, 0.97, and 0.95 were calculated for the X71, X163, and RNAlib libraries in RS media, respectively. For the RIS conditions, correlation coefficients reached 0.997, 0.998, and 0.987 with 8x coverage. We note that our full experiments used more than 100x coverage. Error bars represent standard deviations.

Supplementary Tables

Supplementary Table 1. Vectors used in this study. Table describing all vectors used. Yes: Contains indicated feature. No: Does not contain indicated feature, N/A: Not Applicable

Vector	Available from	Resistance Marker	Domain	Transformation Marker	Yeast Origen of Replication	Gateway Compatible	Homology Region 1 and 2	Screen
**pDONR221/ pENTR221**	Thermo Fisher Scientific	Kanomycin	N/A	N/A	N/A	Yes	N/A	N/A
**pDONR223/ pENTR223**	Thermo Fisher Scientific	Spectinomycin	N/A	N/A	N/A	Yes	N/A	N/A
**pGADT7**	Clontech	Ampicilin	Gal4 Activation Domain	LEU2	2µ	No	No	Y2H & Y3H
**pAW**		Ampicilin	Gal4 Activation Domain	LEU2	2µ	Yes	No	Y2H & Y3H
**pAWH**		Ampicilin	Gal4 Activation Domain	No	2µ	Yes	Yes	rec-Y2H & rec-Y3H
**pGBKT7**	Clontech	Kanomycin	Gal4 Binding Domain	TRP1	2µ	No	No	Y2H
**pBW**		Kanomycin	Gal4 Binding Domain	TRP1	2µ	Yes	No	Y2H
**pBKWH**		Kanomycin	Gal4 Binding Domain	TRP1	No	Yes	Yes	rec-Y2H
**pBSWHhc**		Spectinomycin	Gal4 Binding Domain	TRP1	No	Yes	Yes	rec-Y2H
**pIIIA-MS2-2**	Marvin Wickens	Ampicilin	MS2-Binding stem-loop	ADE2	2µ	No	N/A	Y3H
**pMS22H**		Kanomycin	MS-Binding stem-loop	TRP1	No	No	Yes	rec-Y3H
**pFAB**		Ampicilin	Gal4 Binding Domain/ Gal4 Activation Domain	TRP1	2µ	Yes	Fused	rec-Y2H
**pFAM**		Ampicilin	MS2-Binding stem-loop/ Gal4 Activation Domain	TRP1	2µ	Yes	Fused	rec-Y3H

Supplementary Table 2. Yeast strains used in this study.

Yeast strain	Screen	Available from	Genotype	Reporter genes	Transformation markers	rec-YnH Recombination - selection media (RS)	rec-YnH Recombination-Interaction-Selection media (RIS)	Reference
**Y2HGold**	**rec-Y2H**	Clontech	MATa, trp1-901, leu2-3, 112, ura3-52, his3-200, gal4Δ, gal80Δ, LYS2 : :GAL1$_{UAS}$–Gal1$_{TATA}$–His3, GAL2$_{UAS}$–Gal2$_{TATA}$–Ade2 URA3: : MEL1$_{UAS}$–Mel1$_{TATA}$, AUR1-C MEL1	AUR-1, HIS3, ADE2, MEL1	trp1, leu2	SD/-Trp	Low Stringency: SD/-Trp/Aba, Medium stringency: SD/-Trp/-His/-Ade, High stringency: SD/-Trp/-His/-Ade/AbA*.	Clontech. Nguyen, unpublished
**YBZ-1**	**rec-Y3H**	Marvin Wickens lab	MAT**a,** ura3-52, leu2-3, 112, his3-200, trp1-1, ade2, LYS2 :: (LexAop)-HIS3, ura3 :: (lexA-op)-lacZ, LexA-MS2 coat (N55K)	HIS3, lacZ	trp1, leu2, ura3, ade2	SD/-Trp	Low Stringency: SD/-Trp/-His, Medium-high stringency: SD/-Trp/-His/3-AT*	Hook *et al*. RNA. 2005 Feb; 11(2): 227–233.
							*Note: Different concentrations of Aureobasidin A (AbA) and 3-amino-1,2,4-triazole (3-AT) can be used to achive different degrees of stringency	

Supplementary Table 3. Comparison of liquid-gel and agar plate culture.

Media	Volume for 12 transformations	Recipient	Harvesting	Price
Seaprep liquid-gel	250 mL	1x 5L flask	10 min spin + 5 minutes handling	18 Euros
Agar plates	1 L	4x square BioAssay dishes, 245×245 mm (or 30x10-cm plates)	scraping colonies: about 30 min	50 Euros

Table comparing volume of selection media, quantity and type of recipient needed, protocol and time for colony harvesting, and selection media price between liquid-gel media and agar plates.

Supplementary Table 4. Scalability comparison of liquid-gel and agar plate culture.

Media	Volume for 3 screens with 2 selection media each	Units in one incubator	Harvesting	Price
Seaprep liquid-gel	6x 250 mL = 1.5 L	6x 5L flasks	10 min spin + 15 min handling	108 euros
Agar plates	6x 1L = 6 L	24x square BioAssay dishes, 245×245 mm (or 180x 10-cm plates)	3-4 hours	300 euros

Scalability: Volume, recipient, harvesting time and selection media price for 3 rec-Y3H screenings in two selection media each.

Supplementary Table 5. rec-Y2H versus rec-Y3H.

	Part of the protocol	Step	rec-Y2H	rec-Y3H
I.	Bait and Prey library preparation	1. Batch cloning of libraries	pAWH and pBWH by Gateway	pAWH by Gateway, pMS22H by gibson
II.	Bait and Prey library fusion & Selection in liquid gel	2. Competent yeast	Y2HGold	YBZ-1
		3. Linearisation	N/A	N/A
		4. Batch yeast transformation	N/A	N/A
		5a. Recombination-Selection liquid gel media	N/A	N/A
		5b. Recombination-Interaction-Selection in liquid gel	Low Stringency: SD/-Trp/Aba, Medium stringency: SD/-Trp/-His/-Ade, High stringency: SD/-Trp/-His/-Ade/AbA*	Low Stringency: SD/-Trp/-His, High stringency: SD/-Trp/-His/3-AT*
III.	NGS library preparation	6. Harvest yeast	N/A	N/A
		7. Yeast library DNA isolation	N/A	N/A
		8. Covaris shearing	N/A	N/A
		9. Circularisation	N/A	N/A
		10. R1/R2 PCR	Pr4seq_F_TS_R1/ Pr4seq_R_TS_R2	Pr4seq_F_TS_R1/ Pr4seq_MS22_R2_rev
		11. P5/P7 PCR	N/A	N/A
III.	Paired–end sequencing	12. Library quality control and MiSeq	N/A	N/A
		13. Analysis	N/A	N/A

Table indicating the differences between rec-Y2H and rec-Y3H. N/A No difference between rec-Y2H and rec-Y3H

Supplementary Table 6. Cost of performing a rec-YnH assay.

	Part of the protocol	Step	Quantity	Price	Total
**I.**	**Bait and Prey library preparation**	1. Batch Gateway cloning	4 reactions x 2 pDEST pools	192 Euros	Once for every library. Total 192 Euros
**II.**	**Bait and Prey library fusion & Selection in liquid gel**	2. Competent yeast	1culture	2 Euros	
		3. Linearization	6 reactions x 2 enzymes x 2 pDEST pools	12 Euros	
		4. Batch yeast transformation	12 transformations x 2 selection media	12 Euros	
		5. Selection liquid-gel media	2 selection media	36 Euros	
**III.**	**NGS library preparation**	6. Harvest yeast	2 selection media	N/A	
		7. Yeast library DNA isolation	6 preps x 2 selection media	66 Euros	
		8. Covaris shearing	1 DNA pool x 2 selection media	14 Euros	
		9. Circularisation	1 DNA pool x 2 selection media	16 Euros	
		10. R1/R2 PCR	10 reaction x 2 selection media	32 Euros	
		11. P5/P7 PCR	6 reaction x 2 selection media	18 Euros	For every replicate (with 2 selection media). Total 208 Euros
**IV.**	**Paired –end sequencing**	12. Library quality control and MiSeq	1 run	Service dependent	Service dependent
		13. Analysis		N/A	

Part I. Bait and Prey library preparation is done only once; the resulting libraries can be used for at least 5 to 10 full-screen replicates, with two different selection media each. Costs are calculated for both bait and prey library preparation. Parts II - IV are performed for every replicate and costs are calculated for two selection media (RS and RIS conditions).

Supplementary Methods

Detailed Protocol

rec-YnH enables simultaneous many-by-many detection of direct protein-protein and protein-RNA interactions

Jae-Seong Yang[1,*,#], Mireia Garriga-Canut[1,*], Nele Link[1], Carlo Carolis[1], Katrina Broadbent[1],Violeta Beltran-Sastre[1], Luis Serrano[1,2,3], Sebastian P. Maurer[1,2,#]

Abstract

Knowing which proteins and RNAs directly interact is essential for understanding cellular mechanisms. Unfortunately, discovering such interactions is still resource intensive and comes with considerable uncertainties. With the aim of creating a reliable, low-tech and affordable screen, we developed rec-YnH, a new yeast two- or three-hybrid-based screening pipeline capable of detecting interactions within protein libraries or between protein libraries and RNA fragment pools. rec-YnH combines batch cloning and transformation with intracellular homologous recombination to generate bait-prey fusion libraries with high efficiency. This protocol accompanies Yang, Garriga et al., Nature Communications, 2018.

Introduction

Revealing which proteins, protein domains and RNAs undergo direct, physical interactions is an essential prerequisite for understanding the principles by which c.ellular machines operate. Assays that can detect such interactions are in high demand as they function both to generate system-level views of cellular coordination but also to generate hypothesis for subsequent mechanistic studies[2]. Yeast two and three hybrid (Y2H, Y3H) screens[3,4], allow to detect direct protein-protein and protein-RNA interactions under physiological conditions. The Y2H assay was enhanced significantly in the past. Coupling the readout to next-generation sequencing (NGS)[5] and multiplexed generation of genetically fused bait-prey libraries[1,6] resulted in increased assay sensitivity and reproducibility while allowing screening many baits against many preys simultaneously. Improvements of Y3H, however, mostly focused on optimized RNA binding and presentation by linker RNA engineering and selection of stronger MS2-binding proteins[7]. To date, there is no method available allowing the many-by-many direct interaction detection of full-length RBPs and RNA-fragments.

With the development of rec-YnH, we aimed at creating one assay that further advances and integrates past developments in Y2H and Y3H technologies into a single, new assay pipeline allowing screening of protein libraries against protein libraries or RNA fragment libraries. At the same time, we focused on keeping the assay workflow as simple as possible to make it affordable and doable for the standard biomedical research lab. The assay pipeline combines batch-cloning and transformation with intracellular homologous recombination, liquid gel culturing, yeast two or three-hybrid based interaction detection and read out by next-generation-sequencing. This eliminates barcoding, arraying and plating steps needed for available methods. Three compatible vectors allow high-throughput interaction screening within protein libraries and, for the first time, between protein and RNA libraries. We demonstrate that the same assay pipeline can be used for rec-Y2H and rec-Y3H screening and thus create an assay for both, many-by-many protein-protein or protein-RNA interaction detection.

SECTION 1: SUPPLEMENTARY METHODS FOR rec-Y2H

rec-YnH WORKFLOW

Next, a detailed protocol for rec-Y2H, a protein-protein interactions screening between a library of baits and a library of preys, can be found. rec-Y3H screening, to identify RNA-protein interactions, follows the same protocol as rec-Y2H, with minor differences. A table with differences between rec-Y2H and rec-Y3H can be found in SECTION 2, as well as a detailed description of the steps that are specific for rec-Y3H

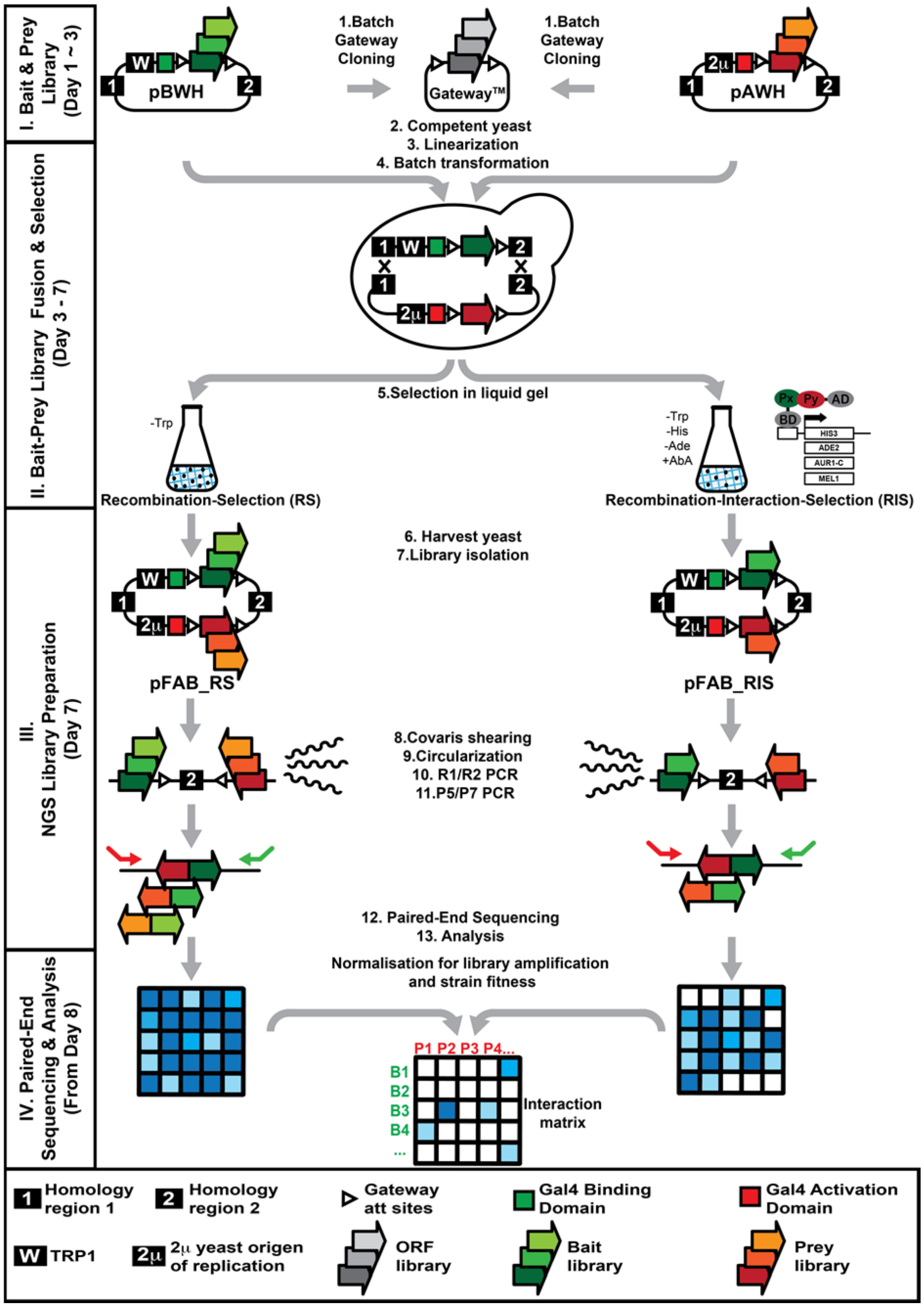

PARTS OF THE PROTOCOL (Figure 1)

Part I. Bait and Prey library preparation (Fig. S3)

Step 1. Batch Gateway cloning of ORF library into pDEST vectors

Part II. Bait and Prey library fusion & Selection in liquid gel (Fig. S5)

Step 2. Competent yeast
Step 3. Linearization
Step 4. Batch yeast transformation
Step 5. Growth in liquid-gel selection media

Part III. NGS library preparation (Fig. S6)

Step 6. Harvest yeast
Step 7. Yeast library DNA isolation
Step 8. Covaris shearing
Step 9. Circularisation
Step 10. R1/R2 PCR
Step 11. P5/P7 PCR

Part IV. Paired-end sequencing (Fig. S8)

Step 12. Library quality control and paired-end MiSeq
Step 13. Analysis

PROTOCOL AT A GLANCE, DAY BY DAY

DAY 1

Step 1. Batch Gateway cloning of ORF library into pDEST vectors
- Prepare pool of pENTR-ORF clones at 10 nM
- Prepare pDEST for Bait and Prey at 10 nM
- Set up LR reactions

Step 2. Prepare competent yeast
- Streak Y2HGold on a YPDA plate

DAY 2

Step 1. Batch Gateway cloning of ORF library into pDEST vectors
- Continue LR reaction
- Transform LR reaction into NEB stable cells
- Plate on LB-Agar with corresponding antibiotic

DAY 3

Step 1. Batch Gateway cloning of ORF library into pDEST vectors
- Colony counting
- Harvest colonies from LB-Agar plates
- Qiaprep DNA prep of Bait and Prey pDEST-ORF pool

Step 2. Competent yeast
- Grow 1 colony of Y2HGold during 8 hours
- Transfer to 50 ml YPDA media
- Grow overnight

Step 3. Linearization
- Digest Bait and Prey pDEST-ORF pool with I-SceI/I-CeuI
- Heat inactivation

DAY 4

Step 2. Competent yeast
- Transfer yeast to 100 ml YPDA
- Grow yeast to OD 0.5
- Wash cells
- Resuspend in TE/LiAc

Step 4. Batch yeast transformation
- Prepare Seaprep liquid-gel selection media
- Multiple transformations of yeast with linear Bait and Prey pDEST-ORF pool
- Pool together all transformations
- Plate dilutions for colony counting

Step 5. Growth in selection media
- Add transformations to liquid-gel selection media
- 1 hour on ice
- Grow at 30ºC for 60 hours

DAY 7

Step 6. Harvest yeast
- Colony counting
- Centrifuge liquid-gel selection media with yeast colonies
- Wash and resuspend in PBS
- OD_{660} to calculate concentration of cells

Step 7. Yeast library DNA isolation
- For each selection media, Zymoprep™ Yeast Plasmid Miniprep of recombined Bait and Prey fragments

Step 8. Covaris shearing
- Shear DNA to 1,500-bp fragments

Step 9. Circularisation
- End repair
- Mini Elute DNA purification
- Ligation
- Heat inactivation

Step 10. R1/R2 PCR
- 12 PCR cycles with Q5 polymerase
- Purification with AMPure beads

Step 11. P5/P7 PCR
- 12 PCR cycles with Q5 polymerase and NEBNext® Multiplex Oligos for Illumina
- Purification with AMPure beads

DAY 8 onwards

Step 12. Paired-end sequencing
- Library quality control
- MiSeq run

Step 13. Analysis

REAGENTS AND MATERIALS

Step 1. Batch Gateway cloning of ORF library into pDEST vectors
- pENTR-ORF clones
- TE buffer (10 mM Tris, pH 8.0, 1 mM EDTA)
- 10 mM Tris, pH 8.0
- LR clonase II enzyme mix (ThermoFisher Scientific)
- NEB stable cells (New England Biolabs)
- SOC media
- 10-cm LB-agar plates
- Ampicillin
- Spectinomycin
- Kanomycin
- 1x PBS
- QIAprep Spin Miniprep Kit (Qiagen)

Step 2. Competent yeast
- Y2HGold (Clontech)
- Loops
- Plastic cuvettes
- YPDA–agar plates
- YPDA liquid media
- 50 ml tubes
- 250 ml flasks
- 500 ml flasks
- Sterile H2O
- TE buffer (10x)
- 1 M LiAc (10x)

Step 3. Linearization
- I-SceI ((New England Biolabs)
- I-CeuI (New England Biolabs)
- Agarose gel

-

Step 4. Batch yeast transformation
- Yeast carrier DNA (Clontech)
- 50% PEG (Sigma)
- 1 M LiAc (10x)
- DMSO (Sigma)
- 0.9% (w/v) NaCl

Step 5. Growth in selection media
Yeast dropout agar plates
- Square BioAssay dishes, 245×245 mm (Corning) for screen
- 10-cm petri dish for colony counting
- Agar (Conda)
- Minimal SD base (Clontech),
- Amino Acid Dropout mixes (Clontech)
- Aureobasidin A (AbA) (Clontech) when indicated

Yeast dropout liquid-gel media
- 5 litre flask
- SeaPrep™ Agarose (Lonza) (0.5% (w/v) final concentration)
- Minimal SD base (Clontech),
- Amino Acid Dropout mixes (Clontech)

- Aureobasidin A (Clontech) when indicated

Step 6. Colony counting and harvesting yeast
- 500 ml centrifuge bottles
- 1xPBS
- Plastic cuvettes

Step 7. Yeast library DNA isolation
- Zymoprep™ Yeast Plasmid Miniprep II (Zymo Research)
- DNAse-free water

Step 8. Covaris shearing
- microTUBE AFA Fiber Pre-Slit Snap-Cap 6x16 mm (covaris)

Step 9. Circularisation
- NEBNext® End Repair Module (New England Biolabs)
- MinElute PCR Purification Kit (Qiagen)
- Quick Ligation™ Kit (New England Biolabs)
- DNAse-free water

Step 10. R1/R2 PCR
- Q5 High-Fidelity 2X Master Mix (New England Biolabs)
- Primer Pr4seq_F_TS_R1 (5'- ccctacacgacgctcttccgatctcgctgcaggtcgacggatc-3') (custom ordered from Integrated DNA Technologies)
- Primer Pr4seq_R_TS_R2 (5'- ttcagacgtgtgctcttccgatctgcagctcgagctcgatggatc-3') (custom ordered from Integrated DNA Technologies)
- Agencourt AMPure XP beads (Beckman Coulter)
- 10 mM Tris, pH 8.0

Step 11. P5/P7 PCR
- Q5 High-Fidelity 2X Master Mix (New England Biolabs)
- NEBNext® Multiplex Oligos for Illumina®, Index Primers Set 1 (New England Biolabs)
- DNAse-free water
- Agencourt AMPure XP beads (Beckman Coulter)
- 10 mM Tris, pH 8.0

Step 12. Paired-end sequencing
- MiSeq Reagent Kit v2 (Illumina)

EQUIPMENT

- PCR machine
- Microcentrifuge
- Clinical centrifuge
- Ultracentrifuge
- Water bath at 30ºC
- Water bath at 42ºC
- Spectrophotometer
- Nanodrop
- Shaker at 30ºC
- Shaker at 37ºC
- Incubator at 30ºC
- Incubator at 37ºC
- Horizontal Electrophoresis System for DNA
- S220 Focused-ultrasonicator with AFA Technology (Covaris)

SECTION 1: SUPPLEMENTARY METHODS FOR rec-Y2H

DAY 1

Step 1. Batch Gateway cloning of ORF library into pDEST vectors

For each screen, both a bait and a prey library need to be built. Therefore, the following steps are performed independently but in parallel for both pDEST-bait and pDEST-prey libraries.

ORF pool	pDEST
**Bait**	pBWH (pBSWHhc or pBKWH)
**Prey**	pAWH

Table of compatibility between pDEST and pENTR

pDEST	pENTR221 (Kanomycin)	pENTR223 (Spectinomycin)
**pAWH (Ampicilin)**	Yes	Yes
**pBSWHhc (Spectinomycin)**	Yes	No
**pBKWH (Kanomycin)**	No	Yes

- Dilute each pENTR-ORF clone to 10 nM in 10 mM Tris, pH 8.0
- Mix together 5 µl of each pENTR-ORF clone at 10 nM to generate a pENTR-ORF pool at a final concentration of 10 nM. We recommend a maximum of 96 clones in each pool (see Note 1 for libraries with more than 96 clones).
- Dilute pDEST to 10 nM in 10 mM Tris, pH 8.0
- Set up 4 Batch Gateway LR reactions

Component	Volumes for 5 µl reaction	x4 reactions
**pENTR-ORF pool 10 nM**	1.5 µl	6 µl
**pDEST 10 nMl**	1.5 µl	6 µl
**TE**	1 µl	4 µl
**LR clonase II**	1 µl	4 µl
	-------	-------
	5 µl	Split in 4x 5 µl

- Incubate 8 hours at 25ºC
- Add to each tube

pDEST 10 nM	1.5 µl
LR clonase II	1 µl

- Incubate 8 hours at 25ºC

Step 2. Competent yeast

- Streak Y2HGold on a YPDA plate

DAY 2

Step 1. Batch Gateway cloning of ORF library into pDEST vectors

- Add to each tube

pDEST 10 nM	1.5 µl
LR clonase II	1 µl

- Incubate 8 hours at 25ºC
- Add 1 µl of Proteinase K to each LR reaction tube
- Incubate 10 minutes at 37ºC (if needed, DNA can be stored at -20ºC at this point)
- For each LR reaction, transform 1 µl into 27 µl of NEB stable cells
- Spread each transformation onto 2x 10 cm LB-agar-antibiotic plates (100 µl on each plate, total of 8 plates). See antibiotic marker in the above table.
- Spread 100 µl of 1/10, 1/100, and 1/1000 dilutions onto 10-cm LB-agar-antibiotic plates to count colonies
- Grow all plates for 16 hours at 37ºC

DAY 3

Step 1. Batch Gateway cloning of ORF library into pDEST vectors

- Count colonies
- Harvest and combine colonies from 8 plates by using 2x 5 ml of PBS for each plate and the aid of a cell spreader
- Make serial dilutions to measure OD
- Use 8 Qiaprep Spin Miniprep columns, with 15 OD units per column to prepare the pDEST-ORF pool, elute with 100 µl of EB buffer and pool together
- Dilute to 5 nM (to calculate the amount (ng) of the pDEST-ORF pool needed to make a 5 nM dilution, consider the average size of all ORFs in that pool)
- Summary

pDEST-ORF pool	# LR reactions	Total # plates per pDEST	Total # Qiaprep spin columns	Final volume of Qiaprep pool	Dilute to
pAWH ORF pool	4	8	8	800 µl	5 nM
pBSWHhc or pBKWH ORF pool	4	8	8	800 µl	5 nM

Step 2. Competent yeast

- In the morning, inoculate 1 fresh colony (2-3 days old) of Y2HGold into 3 ml of YPDA medium
- Grow at 30ºC with shaking at 200 rpm for 8 hours
- Transfer 10, 25 or 50 µl of yeast culture to 50 ml YPDA medium in a 250 ml flask
- Grow at 30ºC with shaking at 200 rpm for 16 hours

Step 3. Linearization

- When building a library containing more than 96 clones, different library sub-pools should be mixed at this stage. See NOTE 2.
- To set up the homing digest for linearization of pDEST-ORF pools, calculate the final amount of linear DNA needed for all transformations. For a typical screen with 96 clones and two types of selection media (recombination-selection (RS) and recombination-interaction-selection (RIS) media), see table below. Scale up accordingly when using larger libraries and/or more than one interaction-selection media. See NOTE 3.

pDEST-ORF pool	□RF poolsee table below. Scale u	# of transformations for a library with 96 clones/ yeast selection media	# of selection medias used	Total volume of homing digest needed	# of 50 □5 homing digest reactions needed (in excess)
**pAWH-ORF pool**	10 µl	12	2	240 µl	6
**pBSWHhc or pBKWH ORF pool**	10 µl	12	2	240 µl	6

- Digest each pDEST (pAWH or pBWH) with I-CeuI and I-SceI to linearize the vector at homology regions 1 and 2, respectively

Component	Volumes for 50 µl reaction	Volumes x6 reactions
**pDEST ORF pool at 5 nM**	20 µl	120 µl
**H2O**	23 µl	138 µl
**NEB Buffer CutSmart**	5 µl	30 µl
**I-SceI**	1 µl	5 µl
**I-CeuI**	1 µl	5 µl
	------	------
	50 µl	split in 6 x 50 µl

- Incubate at 37ºC for 16 hours in a PCR machine block with a heated lid
- Heat inactivate at 65ºC for 20 min

<u>DAY 4</u>

Step 2. Competent yeast

- Take the flasks with Y2HGold from day 3
- Measure OD_{600}, and choose the flask with an OD between 0.3 and 0.4
- Centrifuge 15 OD units of cells at 700 g for 5 min at room temperature
- Resuspend the pellet in 100 ml of YPDA medium and transfer to a 500-ml flask
- Grow at 30ºC with shaking at 200 rpm until OD reaches 0.4-0.5 (approximately 3 hours)
- Centrifuge cells in 2 x 50 ml tubes at 700 g for 5 min
- Wash each pellet with 25 ml of H2O
- cells at 700 g for 5 min
- Resuspend each pellet with 900 µl of 1.1xTE/LiAc solution and transfer to a 1.5 ml eppendorf

- Centrifuge at maximum speed for 30 s
- Resuspend each pellet with 600 µl of 1.1xTE/LiAc solution
- Store in ice (use the same day)

Step 3. Homing digest

- For each pDEST-ORF pool, mix together 6 homing digestion reactions
- Keep on ice

Step 4. Batch yeast transformation

A typical screen requires the libraries to be grown in RS media and RIS media. However, different stringencies for RIS media can be used (see NOTE 3). All the transformations need to be mixed together, and then split into the total number of selection medias to be used. Typically, we use one RS media and one RIS media for each replica.

- Prepare 250 ml of liquid gel RS and RIS media by adding 0.5% Seaprep agarose to the yeast media
- RS media: SD/-W
- RIS media: Choose one or more of the following (see NOTE 3)
- SD/-W/AbA (low stringency)
- SD/-W/-H/-A (medium stringency)
- SD/-W/-H/-A/AbA (high stringency)

  Note: Concentration of Aureobasidin A (AbA) can be varied for further altering stringency
- Autoclave liquid-gel media with a magnetic stirrer
- Stir at room temperature to cool down until for use
- Set up 12 small-scale transformations for each selection media
- Denature yeast carrier DNA at 95ºC for 5 min and chill on ice
- To pre-chilled, sterile tubes, add the following components in order

**Component**	**Volumes for each transformation**
pAWH-ORF pool Homing digest at 2 nM	10 µl
pBSWHhc- or pBKWH-ORF pool Homing digest at 2 nM	10 µl
Denatured yeast carrier DNA	5 µl
Competent Y2HGold	50 µl
PEG/LiAc	500 µl

- Mix by inverting
- Incubate 30 min at 30ºC, inverting tubes every 10 min
- 20 µl of DMSO to each transformation
- by inverting
- 15 min at 42ºC, inverting tubes every 5 min
- Centrifuge 30 seconds at maximum speed
- Resuspend each transformation in 200 µl of 0.9% NaCl

Step 5. Growth in selection media

- Mix together all transformations and measure total volume (approximately 10 ml)
- Take 100 µl to make serial dilutions (1/5, 1/25, 1/125, 1/625) for counting colonies

- Plate 100 µl of each dilution onto 10-cm RS agar plates to calculate total number of transformants screened
- Plate 100 µl of each dilution on 10-cm RIS media agar plates to calculate the percentage of positive interactions.
- Split the total volume of transformed yeast in two (or more if using more than one RIS media) and add to:
- 250 ml Seaprep liquid-gel RS media
- 250 ml Seaprep liquid-gel RIS media
- Stir
- For each liquid-gel selection media, transfer 250 ml to a 5 litre flask
- Place flasks on a tray with ice for 1 hour
- Carefully place flasks in a 30ºC incubator (this incubator should be as stable as possible to avoid the movement of the liquid-gel media)
- Incubate at 30ºC for 60 hours

DAY 7

Step 6. Harvest yeast

- Count the total number of colonies grown on the RS plates and RIS plates
  Total # colonies = # colonies * dilution factor * total volume / plating volume
  # colonies: number the colonies that appear on the plate
  Dilution factor: dilution used on the plate
  Total volume: final volume (in ml) in which the yeast cells were resuspended and added to one selection media
  Plating volume (in ml): if 100 µl were plated on a counting plate, the plating volume is 0.1
- Transfer 250 ml of Seaprep media with yeast colonies to a 500-ml centrifuge bottle
- Centrifuge cells at 1600 g for 10 min
- Wash pellet with 40 ml PBS and transfer to a 50-ml tube
- Centrifuge cells at 700 g 5 min
- Resuspend cells in 40 ml PBS
- Make serial dilutions and measure OD660 to determine the number of cells per ml. For calculations, use the dilution with an OD between 0.3 and 0.5

Step 7. Yeast library DNA isolation

For each selection media, prepare DNA independently and in parallel. Recombined yeast plasmid DNA for each selection media is referred to as pFAB_RS and pFAB_RIS
- Calculate the volume of cells in PBS needed to have $4x10^7$ cells
- Use 6 columns, with $4x10^7$ cells on each column, of Zymoprep™ Yeast Plasmid Miniprep kit
- Elute each column with 10 µl H_2O
- Pool together yeast DNA from 6 columns (about 55 µl)

Step 8. Covaris shearing

- Transfer DNA to a microTUBE-50 AFA Fiber (covaris)
- Shear DNA to 1,500 bp by Covaris ultra-sonication under the following conditions: duty cycle, 2%; intensity, 5; cycles per burst, 200; time, 25 seconds
- Save a 3-µl aliquot for running on a bioanalyzer to confirm the DNA quality

Step 9. Circularisation

- End repair

Component	Volumes for 60-µl reaction
Covaris-sheared yeast DNA	51 µl
10x NEBNext End repair buffer	6 µl
NEBNext End repair enzyme	3 µl
	----------
	60 µl

- Incubate at 20ºC for 30 min
- Purify DNA with a MiniElute PCR Purification Kit column
- Elute with 10 µl H_2O
- Intramolecular ligation

Component	Volumes for 20-µl reaction
2xbuffer	10 µl
covaris/End repair	9 µl
Quick ligase NEB	1 µl
	-------
	20 µl

- Incubate 5 min at 25ºC
- Heat inactivate for 20 min at 65ºC

Step 11. R1/R2 PCR:

- Set up 10 PCR reactions of 25 µl each

Component	Volumes for 25 µl reaction	x10 PCR each
Q5 High-Fidelity 2X Master Mix	12.5 µl	125 µl
Nuclease-Free Water	8 µl	80 µl
10 µM Pr4seq_F_TS_R1 (IDT)	1.25 µl	12.5 µl
10 µM Pr4seq_R_TS_R2 (IDT)	1.25 µl	12.5 µl
Template DNA 2 µl	2 µl	20 µl
	----	------
	25 µl	Split in 10x 25 µl

Step	Temp	Time
Initial Denaturation	98°C	30 seconds
12 Cycles	98°C	10 seconds
	72°C	60 seconds
Final Extension	72°C	5 minutes

- Pool together 10 PCR reactions (at this stage, DNA can be stored at -20ºC for further use)
- Measure volume (approximately 200 µl)
- Split sample in 50 µl aliquots (typically 4 aliquots for each selection media)
- Add 35 µl of AMPure XP beads to each 50 µl aliquot of R1/R2 PCR
- Incubate 5 min
- Quickly spin the tube (no more 400 g for 5 seconds)
- Place tubes on a magnetic rack
- After the solution is clear (5 min), carefully remove and discard the supernatant. Do not disturb the beads.
- Wash 3x 200 µl with 80% ethanol
- With the tubes on the magnetic rack, air dry the beads for 10 min
- Elute all aliquots from one same selection media with a total volume of 30 µl 10 mM Tris, pH 8.0 (by transferring beads and buffer from one aliquot to the next)
- Let the tube stand for 5 min at room temperature
- Quickly spin the tube (no more 400 g for 5 seconds)
- Place tubes on the magnetic rack
- After the solution is clear (5 min), carefully take 25 µl of supernatant containing the purified DNA

Step 12. P5/P7 PCR

- For each selection media, use a different index primer for multiplexing. Set up 6 PCR reactions of 25 µl each

Component	Volumes for 25 µl Reaction	6 replicates each => x6
Q5 High-Fidelity 2X Master Mix	12.5 µl	75 µl
Nuclease-Free Water	6 µl	36 µl
Template DNA	4 µl	24 µl
10 µM NEBNext Index Primer for Illumina	1.25 µl	7.5 µl
10 µM NEBNext Universal PCR Primer for Illumina	1.25 µl	7.5 µl
	-------	-------
	25 µl	Split in 6x 25 µl

Step	Temp	Time
Initial Denaturation	98°C	30 seconds
Cycles 12	98°C	10 seconds
	65°C	10 seconds
	72°C	45 seconds
Final Extension	72°C	5 minutes

- Pool together 6 PCR reactions
- Measure volume (typically 120 µl)
- Split sample in 50-60-µl aliquots
- Add 30 µl beads per 50 µl of each aliquot of R1/R2 PCR
- Incubate 5 min
- Quickly spin tubes (no more than 400 g for 5 seconds)
- Place tubes on magnetic rack
- After the solution is clear (5 min), carefully remove and discard the supernatant. Do not disturb the beads
- Wash 2x 200 µl with 80% ethanol
- Air dry 10 min
- For each selection media, elute all tubes with a final volume of 30 µl of 10 mM Tris, pH 8.0
- Recover 25 µl (at this stage, DNA can be stored at -20ºC, if needed)
- Run 2 µl of purified PCR product in a 0.8% gel to check quality. A smear of sizes between 600 and 1,200 bp should be seen. The presence of discrete bands indicates biased amplification of some fragments, and a bad quality of the final product

DAY 8 ~

12. MiSeq sequencing

To identify those protein (or RNA) pairs selected for in the RS or RIS media, next generation sequencing is performed in the 2x150-bp paired-end reads mode. We used MiSeq sequencing simply because of its fast turnaround time. It gives about a 10 million read depth, and in our experiments, we generally used over 100 x sequencing depth of all theoretical possible pair-wise combinations. However, with simulation results, over 8x sequencing depth of all possible combinations should be enough to get reliable results (**Supplementary Fig. 17**).

- Analyse DNA after covaris, R1/R2 PCR and P5/P7 PCR with High Sensitivity DNA Analysis kit and bioanalyser.
- Quantify by qPCR
- 2x150-bp paired-end sequencing with MiSeq Reagent Kit v2

13. Processing of sequencing data

Below is a brief description of how the pipeline processes the raw data. Inputs to the pipeline are FASTQ files from one sequencing run and the FASTA file for input proteins or RNAs.

- Reads are trimmed by cutadapt (v.1.14) with the proper common sequences depending on whether it is a PPI or RPI experiment.

- For the protein part, the trimmed read sequences are aligned to the reverse complement 3' coding sequence (for the last 100 nts) of the target library using the blastn (v.2.3.0) program with the blastn-short option and an E-value cut-off of 1e–8.
- For the RNA part, due to the short length of the some of RNAs (< 20nt), an exact motif search is conducted.
- Reads are considered usable reads only if the pair of reads are mapped to target proteins or RNAs.
- PPI/RPI pair reads, are first normalized by the all useable read counts to make frequency matrixes
- Frequency matrixes obtained from RS media are further converted into a null matrix by multiplying marginal distributions of row and column.
- fitting Gaussian mixture models, noise is removed from the RIS media frequency matrixes.
- By dividing the noise-filtered frequency matrix with a null matrix, an interaction score matrix (IS) is generated.
- If more than one experiments is performed, the IS matrixes are averaged to generate an average IS.
- To reduce the basal auto-activation signal, the upper quartile of the average IS for all baits is subtracted from the average IS scores.

<u>NOTES</u>

NOTE 1: When screening a library with more than 96 ORFs, split the library into several sub-pools, each with a maximum of 96 ORFs (pENTR-ORF pool A, B, C, etc…)

NOTE 2: When building a library with more than 96 clones, multiple pDEST-ORF pools are generated (pDEST-ORF pool A, B, C…). To obtain the final pDEST-ORF pool, sub-pools can be mixed according to the table below:

	**# clones in pool**	**Concentration**	**Mix**
**pAWH-ORF pool A**	X clones	5 nM	X µl
**pAWH-ORF pool B**	Y clones	5 nM	Y µl
**pAWH-ORF pool C**	Z clones	5 nM	Z µl

Final Concentration of pDEST-ORF pool: 5 nM
Final µl of pDEST-ORF pool: X+Y+Z µl

NOTE 3: A typical screen requires the growth of the libraries in RS media and RIS media. However, different stringencies for interaction-selection media can be employed by using different concentrations of Aureobasidin A and/or selecting for more than one reporter gene (e.g., media lacking histidine and adenine).

SECTION 2: SUPPLEMENTARY METHODS FOR rec-Y3H

For RNA-protein interaction screening, rec-Y3H is used. It follows the same protocol as for rec-Y2H, except for the minor differences detailed in the following table. N/A: no difference between rec-Y2H and rec-Y3H. A detailed protocol for these rec-Y3H-specific steps are given below.

	Part of the protocol	Step	rec-Y2H	rec-Y3H
**I.**	**Bait and Prey library preparation**	1. Batch cloning of libraries	pAWH and pBWH by Gateway	pAWH by Gateway, pMS22H by gibson
**II.**	**Bait and Prey library fusion & Selection in liquid gel**	2. Competent yeast	Y2HGold	YBZ-1
		3. Linearisation	N/A	N/A
		4. Batch yeast transformation	N/A	N/A
		5a. Recombination-Selection liquid gel media	N/A	N/A
		5b. Recombination-Interaction-Selection in liquid gel	Low Stringency: SD/-Trp/Aba, Medium stringency: SD/-Trp/-His/-Ade, High stringency: SD/-Trp/-His/-Ade/AbA*	Low Stringency: SD/-Trp/-His, High stringency: SD/-Trp/-His/3-AT*
**III.**	**NGS library preparation**	6. Harvest yeast	N/A	N/A
		7. Yeast library DNA isolation	N/A	N/A
		8. Covaris shearing	N/A	N/A
		9. Circularisation	N/A	N/A
		10. R1/R2 PCR	Pr4seq_F_TS_R1/ Pr4seq_R_TS_R2	Pr4seq_F_TS_R1/ Pr4seq_MS22_R2_rev
		11. P5/P7 PCR	N/A	N/A
**III.**	**Paired–end sequencing**	12. Library quality control and MiSeq	N/A	N/A
		13. Analysis	N/A	N/A

rec-Y3H SPECIFIC REAGENTS AND MATERIALS

- YBZ-1
- Primer Pr4seq_MS22_R1_rev
  - (5' ccctacacgacgctcttccgatctgcaggcatgcaagctgcc -3')
- Oligo_giv_rev
  - (5'-CAGGCATGCAAGCTG-3')

DETAILED PROTOCOL FOR rec-Y3H AND SPECIFIC STEPS

Step 1. Gibson cloning of RNA sites

- For each RNA site, a single-stranded oligonucleotide with Gibson overhangs is ordered, where XXX represents an RNA motif.
  5'-gaactagtggatcccXXXccgggcagcttgcatgcctg-3'
- Set up annealing reaction

	25-µl reaction	Final concentration
**RNA site oligonucleotide**	0.5 µl	1 µg
**Oligo_giv_rev**	0.5 µl	0.2 µg
**1x NEB buffer 2.1**	2.5 µl	1x
**H2O**	21.1 µl	

- Heat at 95°C for 2 min
- Slowly cool down
- Add:

	25-µl reaction	Final concentration
dNTPs 10 mM	0.1 µl	33 µM
DNA Polymerase I, Large (Klenow) Fragment 5 U per µl	0.3 µl	1 U per µg DNA

- Incubate 15 min at 25°C
- Purify DNA with a MiniElute PCR Purification Kit column
- Elute DNA with 10 ul of H_2O
- Cut 1 µg of pMS22KH with XmaI for 1 hour at 37°C
- Run on a 0.7% agarose gel
- Cut the band corresponding to the linearized vector
- Purify with QIAquick Gel Extraction kit using one Minielute column
- Elute with 10 µl Elution buffer
- Set up Gibson reaction:

Klenow filled-in DNA	1 µl
XmaI linearized pMS22KH vector	1 µl
Gibson mix (CRG facility)	10 µl

- Incubate 1 hour at 50°C
- Transform 2 µl of Gibson reaction into 50 µl of Stellar Competent Cells (Clontech)
- Pick one colony for each RNA site
- Prepare plasmid DNA with Qiagen spin prep
- Dilute each pMS22H RNA motif to 5 nM
- Mix equal amounts of each pMS22H-RNA, to obtain an equimolar pMS22H-RNA pool with a final concentration of 5 nM

Step 2. Competent yeast

Same as for rec-Y2H, but the YBZ-1 yeast strain is used instead of Y2HGold

Step 5. Growth in selection media

Recombination media is SD/-Trp, as for rec-Y2H
Recombination-interaction-selection media is:

- Low Stringency: SD/-Trp/-His
- Medium-high stringency: SD/-Trp/-His/3-AT*

Step 11. R1/R2 PCR

Same as for rec-Y2H Gold, but use the Pr4seq_MS22_R1_rev primer instead of Pr4seq_R_TS_R1.

Supplementary References

1 Yachie, N. *et al.* Pooled-matrix protein interaction screens using Barcode Fusion Genetics. *Molecular systems biology* **12**, 863, doi:10.15252/msb.20156660 (2016).

2 Fessenden, M. Protein maps chart the causes of disease. *Nature* **549**, 293-295, doi:10.1038/549293a (2017).

3 SenGupta, D. J. *et al.* A three-hybrid system to detect RNA-protein interactions in vivo. *Proceedings of the National Academy of Sciences of the United States of America* **93**, 8496-8501, doi:10.1073/pnas.93.16.8496 (1996).

4 Fields, S. & Song, O. A novel genetic system to detect protein-protein interactions. *Nature* **340**, 245-246, doi:10.1038/340245a0 (1989).

5 Weimann, M. *et al.* A Y2H-seq approach defines the human protein methyltransferase interactome. *Nature methods* **10**, 339-342, doi:10.1038/nmeth.2397 (2013).

6 Trigg, S. A. *et al.* CrY2H-seq: a massively multiplexed assay for deep-coverage interactome mapping. *Nature methods* **14**, 819-825, doi:10.1038/nmeth.4343 (2017).

7 Wurster, S. E. & Maher, L. J., 3rd. Selections that optimize RNA display in the yeast three-hybrid system. *RNA* **16**, 253-258, doi:10.1261/rna.1880410 (2010).
