# Supplementary material for: rec-YnH enables simultaneous many-by-many detection of direct protein–protein and protein–RNA interactions
Source: Nat Commun. 2018 Sep 14;9:3747. doi: 10.1038/s41467-018-06128-x (PMC6138660; doi:10.1038/s41467-018-06128-x)
Supplement: Supplementary file 3 — Description of Additional Supplementary Files [file 41467_2018_6128_MOESM3_ESM.pdf]

Description of Additional Supplementary Files

File Name: Supplementary Data 1
Description: List of X76 input protein library

File Name: Supplementary Data 2
Description: Read mapping statistics

File Name: Supplementary Data 3
Description: List of X76 interaction pairs above cut-off screen results

File Name: Supplementary Data 4
Description: Individual spot-test results and performance comparisons

File Name: Supplementary Data 5
Description: List of X163 input protein library

File Name: Supplementary Data 6
Description: List of X163 interaction pairs above cut-off screen results

File Name: Supplementary Data 7
Description: List of RNAlib input library
